# Destructive and optical non-destructive grape ripening assessment: Agronomic comparison and cost-benefit analysis

**DOI:** 10.1371/journal.pone.0216421

**Published:** 2019-05-29

**Authors:** Sara Savi, Stefano Poni, Alessandro Moncalvo, Tommaso Frioni, Irene Rodschinka, Linda Arata, Matteo Gatti

**Affiliations:** 1 Department of Sustainable Crop Production, Università Cattolica del Sacro Cuore, Piacenza, Italy; 2 Department of Agricultural and Food Economics Università Cattolica del Sacro Cuore, Piacenza, Italy; Universita degli Studi di Siena, ITALY

## Abstract

Reliability and economic assessment of the Multiplex^®^ optical sensor employed for non-destructive grape parameters estimates was evaluated in 2017 against a wet chemistry approach in mature vineyards including five cultivars (two whites, two red and one pink colored) assuring a broad range of variation in both technological and phenolic maturity parameters. Among the five Multiplex (Mx) indices evaluated (SFR_R, FLAV, FLAV-UV, ANTH_RG and FERARI) in all cultivars with the exception of Barbera SFR_R showed a significant and linear correlation with total soluble solids (TSS) for TSS ≥ 10 °Brix. Conversely, no significant correlation was found between FLAV and total flavonols concentration, whereas a significant linear correlation was found in Barbera (R^2^ = 0.66) and Ervi (R^2^ = 0.63) when the FLAV index was replaced with the FLAV_UV index. Within each cultivar, both ANTH_RG and FERARI showed close correlations with total anthocyanins concentration determined by wet chemistry although under different model shapes. Expressing berry color accumulation on a per skin mass basis rather than for whole berry mass basis, allowed for better separation of behavior of single cultivars and improved accuracy of model fitting for the combination of Barbera and ANTH_RG. A strict linear correlation was always found, within each index, for Mx readings taken on the two opposite sides of the same cluster, implying no significant within-cluster differences in sugar, color and flavonol concentrations. Economic assessment of Mx by means of the Net Present Value (NPV) approach showed that Mx is economically viable for a two hectare vineyard cultivating three red grape varieties (90 samples per year) if its lifetime is at least 7 years. Conversely, if only two red varieties are grown Mx should be used at least 11 years to make it economic suitable. Bigger properties growing a higher number of red varieties are the more interested in Mx as the expected NPV assumes positive values with a Mx usage of minimum 3 years.

## Introduction

Literature provides quite robust evidence that, in grapevine red cultivars, the measure of berry color acts as a good predictor for final wine quality [1]. Most of this work was conducted across different climatic regions of Australia on a number of cultivars (primarily Shiraz, Merlot and Cabernet Sauvignon) and overall it was found a quite close correlation between berry color given as total anthocyanin concentration (mg/g of fresh mass) and a wine flavor intensity score [2,3]. However, the same work also indicated that, in Shiraz, by far the most investigated genotype, the color versus wine quality linear correlation was the strongest in the range of 0.4 to 1.6 mg/g of total anthocyanins and that for color values exceeding the higher end up to 2.2 mg/g, the correlation became curvilinear. Likely then, until a certain extent, color increase is associated with an increase of other positive berry flavor components leading to an improvement of the quality of the resultant wine, whereas additional color gains, especially when taking place primarily as a result of berry shrivel, have often led to changes in berry aroma that might not be desirable for the intended wine style [4]. Clearly, especially in warm or hot environments, berry shriveling causes a factitious increase in color concentration while the intensity of overripe character in berries (and wines) is also enhanced and the resultant wine classification may decrease [5].

Despite some objective limitations of the red color vs. wine quality relationship (i.e. the correlation needs to be tested separately for each variety, region and wine style and calibration should be narrowed to low to mid color ranges), such knowledge has fostered over the last years the testing of non-destructive methods for field assessment for anthocyanin concentration in berries and clusters [6–11]. Potential advantages of such approach as compared to the traditional destructive wet chemistry protocols cannot be denied and mainly consist of: i) ability to measure large size samples in a quite limited amount of time also allowing, when needed, a higher frequency of readings throughout the season: ii) readings can be repeated over time on the same individuals therefore diminishing, for instance, the effect due to between-vines and within-plant variability and iii) due to their non-destructive nature, these methods overcome a classic bias of destructive approaches, that is ripening dynamic of the pending crop might be significantly altered as function of the amount of removed berries for sampling, especially when reiterated on the same vines.

Among available technologies for non-destructive assessment of grape ripening, a great interest was risen when the Multiplex^®^ sensor (FORCE-A, Orsay, France), a no contact, hand-held optical device, was launched [11]. The measuring principle for the equipment is based on the interference effect exerted by anthocyanins (ANTH hereafter) absorbance over chlorophyll fluorescence excitation in the green and red spectral regions [7]. The sensor allows calculation of two specific ANTH indices: the ANTH_R based on a single signal, the far-red chlorophyll fluorescence excitation under red excitation, also termed FERARI (Fluorescence Excitation Ratio Anthocyanins Relative Index) after [12] and the ANTH_RG based on two signals, i.e. the ratio between far-red chlorophyll fluorescence under red and under green light excitation. Since Mx introduction, a large number of papers [13–17] has been published to evaluate the potential of the above indices for assessing temporal and spatial variability of color in a number of red cultivars largely differing in their natural attitude to accumulate anthocyanins and varying from poor coloring varieties such as Pinot noir and Nebbiolo (the tested maximum ANTH concentration reaching 0.9 mg/g) to the highest 2.5 mg/g scored by Shiraz and Cabernet Sauvignon [15]. Prediction models based on the ANTH_R and ANTH_RG and calibrated against wet chemistry data were overall good or very good; however, these models were largely obtained from single year determinations and differed as a function of cultivar and, recently, the dependence of the ANTH_RG index on the cultivar Anth profile [16] was shown. Then, for a given cultivar, it is well known that total amount of anthocyanins and single anthocyanidin forms can greatly vary according to site, vintage and cultural practices [18]. For instance, vintage year and site can greatly affect color accumulation through their direct effects on vigor and yield. It is well accepted that color in red cultivars is a function of the leaf area-to-yield ratio (LA/Y), usually levelling off when LA/Y reaches the saturating threshold of 1.2–1.5 m^2^/kg [19]. ANTH accumulation is even more sensitive to local cluster microclimate and it has been shown that training systems [20] or cultural practices [21] causing excessive light exposure, hence overheating, lead to poor coloration or even a decay of previously accumulated color [22]. The recent paper by Pinelli et al. (2018) was the first attempt to address dependence of the prediction models on cultivar, site and year. Their extensive data set included four cultivars, two sites and three seasons and showed that a global (i.e. inter-cultivar and inter-site) calibration model can be used for the prediction of total ANTH concentration in grape berries, with relative errors no higher than 14.1% and 19% for ANTH_R and ANTH_RG, respectively, depending on both the cultivar and the growing site. Though, when seasonal variability was taken into account by pooling data over cultivar and sites, the magnitude of the relative error increased varying from 26% to 33% for ANTH_R and from 17% to 22% for ANTH_RG.

Although the above scenario is in favor of a good adaptability of the two specific ANTH indices derived from Mx measurement to a number of vineyard conditions, wider adoption of the equipment should also consider that narrowing its use to prediction of color development might be perceived as a limiting factor. As for any quite costly equipment introduced in the vineyard, the customer would appreciate it to be multi-usable or multitasking (i.e. largest amount of information obtained from the same apparatus used under different machine settings, suitability to perform prescription color mapping in anticipation of a selective harvesting approach, etc.). While it is indeed ascertained that color is, within certain limits, a good predictor of final wine quality, it is also true that final-price scores of grape loads delivered to the winery are still primarily dependent upon total soluble solids concentration (°Brix) and, occasionally, on health status. Moreover, several recent papers have reported that, fostered by climate change, sugar and color accumulation tend to de-couple along the season especially in warm environments (e.g. high potential alcohol content might be already reached mid-August when flavonoid accumulation is still lagging behind) and this behavior urges that seasonal trends of both components should be tracked [23–27]. Moreover, some grape components such as flavonols, and namely quercetin-3-glucoside have proven to act as sunscreen compounds during ripening and their concentration can increase by several folds with increased light exposure of the berry or cluster [28]. Flavonols are so reactive to solar radiation that they can almost be considered as “light biosensors” and knowing their concentration in berries can aid a lot in determining the most suited harvest time [18]. Mx configuration, as a matter of fact, allows the calculation of two more indices named FLAV [29] and SFR_R [12] that previous work has shown to be quite closely correlated to destructive determinations of total flavonols and TSS concentration, respectively [16].

While a number of studies have been published on calibration and validation of Mx indices over different cultivars, seasons and environments, we are aware of no contributions dealing with its economic assessment versus the traditional approach of manual field sampling and wet chemistry analysis in the laboratory.

This paper aims at the main following objectives: i) provide a full comparison on a range of five cultivars (two whites, two reds and one pink colored) of seasonal trends and correlations for total soluble solids (TSS), total flavonols and total ANTH determined by wet chemistry and Mx; ii) determine, in the colored varieties, the incidence of the relative skin growth and assess if expressing ANTH concentration on whole berry or skin mass would significantly affect the correlation with the Mx ANTH indices and iii) provide economic assessment of Mx by means of the Net Present Value (NPV) approach.

## Materials and methods

### Plant material and growing site

The trial was done in 2017 at the Mossi1558 Estate sited in Ziano Piacentino (225 a.sl., 44° 26 N, 9° 42’ E), Piacenza, Italy. The study was conducted on private land and the owner gave permission to carry it out. The farm is comprised of 55 hectares of vineyards including all major varieties grown in the Colli Piacentini appellation area (e.g. Barbera, Croatina, Ortrugo and Malvasia di Candia aromatica). The study was conducted on five cultivars, two whites (Ortrugo and Malvasia di Candia aromatica), two reds (Barbera and Ervi) and on one with dark-pink colored berries, named Malvasia Rosa (Malvasia R.), originating from a bud sport of Malvasia di Candia aromatica (Malvasia C.a.). Ervi is a new cultivar obtained by crossing Barbera and Croatina, showing very high color accumulation rates, improved traits as compared to its parents, and high adaptability to vineyard mechanization. The five cultivars were spread into different plots; three of them (Ortrugo, Malvasia R. and Ervi) were in an experimental collection vineyard of 0.4 ha planted 2.2m x 2m (between row and within row spacing, respectively, with paired vines) yielding a total of 4545 vines/ha. Barbera and Malvasia C.a. were located in adjacent vineyards planted at 2.5m x 1m, (between row and within row spacing, respectively) resulting in 4000 vines/ha. All vineyards were quite similar in age (10 to 15 years), trained to a single cane horizontal Guyot raised 90cm above ground and provided with upper foliage wires so that final canopy height was around 1.4–1.5 m. In all cases row orientation was NW-SE (i.e. 160° as measured clockwise from North). For each cultivar, a second experimental factor was introduced by taking non-destructive grape quality assessment readings on both sides of each measured cluster (i.e. South-West and North-East facing).

### Optical sensor readings and indices

The Multiplex (Mx) fluorimetric sensor (FORCE-A, Orsay, France) is described in detail by Ben Ghozlen et al. [12]. The Mx uses light-emitting diodes (LED) and filtered photodiodes for fluorescence excitation and detection, respectively. Fluorescence detection is synchronized to the pulsed excitation light for field work under natural light conditions on leaves and fruits. The wide detection area of the sensor (8-cm diameter) makes it possible for a signal to be acquired from a large area in each cluster. Four indices were used here:

$$\begin{array}{ll} \text{ANTH}\_\text{RG}=\text{log}\left(\text{FRF}\_\text{R}/\text{FRF}\_\text{G}\right) & \;\text{Agati}\;\text{et}\;\text{al}.\;\left(2007\right) \end{array}$$

$$\begin{array}{ll} \text{FERARI}=\text{log}\left(1/\text{FRF}\_\text{R}\right) & \;\text{Ben}\;\text{Ghozlen}\;\text{et}\;\text{al}.\;\left(2010\right) \end{array}$$

$$\begin{array}{ll} \text{FLAV}=\text{log}\left(\text{FRF}\_\text{R}/\text{FRF}\_\text{UV}\right) & \;\text{Agati}\;\text{et}\;\text{al}.\;\left(2011\right) \end{array}$$

$$\begin{array}{ll} \text{FLAV}\_\text{UV}=\text{log}\left(1/\text{FRF}\_\text{UV}\right) & \;\text{Ferrandino}\;\text{et}\;\text{al}.\;\left(2017\right) \end{array}$$

$$\begin{array}{ll} \text{SFR}\_\text{R}=\text{FRF}\_\text{R}/\text{RF}\_\text{R} & \;\text{Ben}\;\text{Ghozlen}\;\text{et}\;\text{al}.\;\left(2010\right) \end{array}$$

where FRF_R, FRF_G and FRF_UV are far-red fluorescence under red, green and UV excitation, respectively. RF_R is red fluorescence under red excitation. In addition to the four indices reported above, the FLAV_UV index was also calculated as log (1/FRF_UV). Mx fluorescence signals were corrected for residual electronic offsets and normalized to a fluorescence standard (blue plastic foil, FORCE-A).

### Non-destructive and destructive sampling

For each variety, three rows were chosen and four vines were randomly tagged. For each vine, three clusters to represent basal, median and distal positions along the horizontal cane were marked and, to provide proper calibration of the Mx apparatus, each cluster was optically measured. The operator quickly took two readings per cluster, with the sensor placed in front of the two opposite cluster sides facing South-West and North-East. For each cultivar, these measures were repeated 8 times, at weekly intervals during the time window comprised between onset of veraison (i.e. TSS around 5 °Brix) and pre-harvest. On the same dates, about 500 g of fresh mass was taken from each of three different sections of each row (low, median and high along a gentle slope) having accuracy not to sample berries from the vines tagged for Mx readings. Samples were quickly taken to the laboratory for subsequent wet chemistry analysis. Ripening curves for total soluble solids, titratable acidity, malic acid and total anthocyanin concentration were built over season according to the methods described below. At each sampling date from the same 500 g fresh mass, a 40-berry subsample was taken and immediately frozen at -20 °C for subsequent determination of individual berry organ fresh mass according to the methodology described in the next section. Likewise, a 25-berry sample was taken for subsequent total anthocyanin and phenolic analyses.

At harvest, occurring on DOY 233 (August 21) for Malvasia C.a. and Ortrugo and on DOY 249 (September 6) for Malvasia R., Barbera and Ervi, the same tagged clusters subjected to seasonal Mx readings were individually picked, weighted *in situ* and taken to the laboratory where the two sides of each cluster were measured with Mx. Then, all berries per cluster were counted by separating berries from the two opposite cluster sides.

### Wet chemistry

From each grape sample taken at harvest, three sub-samples were collected and subjected to the following laboratory processing. A first 50-berry sample was immediately crushed and the resulting juice was filtered and analyzed to characterize its technological maturity. TSS were determined with a temperature compensated digital refractometer (ATAGO DBX-55, Tokyo, Japan), pH was assessed with a pH-meter CRISON GLP 22 (Crison, Barcelona, Spain). A compact titrator CRISON was used to measure titratable acidity (TA) by titrating the juice with 0.1N NaOH to an end point of 8.2 pH; results were expressed as g/L tartaric acid equivalent. A second 20-berry sample was frozen at -20°C and used for subsequent determination of single berry components (skin, flesh and seeds). Berries were individually weighed and then sliced in half with a razor blade, the seeds and flesh carefully removed from each berry half using a small metal spatula without rupturing any pigmented hypodermal cells, and the seeds carefully separated by hand from the flesh. Both skins and seeds were rinsed in deionized water, blotted dry, and weighed. The skins were stored and lyophilized for the anthocyanin and phenolic determination by HPLC.

On a third sub-sample consisting of 50 healthy berries anthocyanins and total phenols were determined according to [1]. Anthocyanins were measured only in Malvasia R., Barbera and Ervi. Berries were homogenized at 20.000 rpm with an Ultra-Turrax (Rose Scientific Ltd., Edmonton, Canada) homogenizer for 1 min, then 2 g of the homogenate was transferred to a pre-tared centrifuge tube, enriched with 10 mL aqueous ethanol (50%, pH 5.0), capped and mixed periodically for 1 hr before centrifugation at 959 × *g* for 5 min. A portion of the extract (0.5 mL) was added to 10 mL 1 M HCl, mixed and let stand for 3 hr; absorbance was then measured at 520 nm and 280 nm on a JascoV-530 UV spectrophotometer (Jasco Analytical Instruments, Easton, MD, USA). Total anthocyanins and phenolics were expressed as mg per g of fresh berry and skin mass.

### HPLC analyses

Anthocyanins and flavonols were analyzed as described in [30]. Phenolic compounds were extracted from skins after Downey and Rochfort [31]: 0.100 g of freeze-dried skin were extracted in 1.0 mL of 50% (v/v) methanol in water for 15 min with sonication. To quantify organic acids juice was directly injected after filtration through a 0.22 μm polypropylene filter for HPLC while the pre-harvest samples were diluted 4 times before filtration and then transferred to HPLC auto-sampler vials.

For this analysis an Allure Organic Acid Column, 300 × 4.6 mm, 5 μm (Restek, Bellefonte, PA, USA) was used. Separation was performed in isocratic conditions using water, pH adjusted at 2.5 using ortho-phosporic acid. The column temperature was maintained at 30 ± 0.1°C, 15 μL of sample was injected. The elution was monitored at 200–700 nm and detection by UV-Vis absorption with DAD at 210 nm. Organic acids were identified using authentic standards and quantification was based on peak areas and performed by external calibration with standards.

The chromatographic method was developed using an Agilent 1260 Infinity Quaternary LC (Agilent Technology, Santa Clara, CA, USA) consisting of a G1311B/C quaternary pump with inline degassing unit, G1329B autosampler, G1330B thermostat, G1316B thermostated column compartment and a G4212B diode array detector fitted with a 10 mm path, 1 μL volume Max-Light cartridge flow cell. The instrument was controlled using Agilent Chemstation software version A.01.05. Total flavonols concentration was given as mg/g dry skin mass.

### Chemicals

All solvents were of HPLC grade. Water Milli-Q quality, acetonitrile and methanol were obtained from VWR (Radnor, PA, USA). Formic acid, sodium hydroxide, ortho-phosphoric acid 85%, L-(+)-tartaric acid, L-(-)-malic acid, citric acid, (+)-catechin, (-)-epicatechin, were purchased from Sigma-Aldrich (St. Louis, MO, USA). The following commercial standards from Extrasynthese (Genay, France) were used: *t*-resveratrol, *t*-piceid, myricetin (Myr), myricetin 3-O-glucoside (Myr 3-O-glc), quercetin 3-O-glucuronide (Quer 3-O-glu), quercetin 3-O-glucoside (Quer 3-O-glc), kaempferol 3-O-glucoside (Kmp 3-O-glc), delphinidin 3-O-glucoside (Dp 3-O-glc), cyanidin 3-O-glucoside (Cy 3-O-glc), petunidin 3-O-glucoside (Pt 3-O-glc), peonidin 3-O-glucoside (Pn 3-Oglc) and malvidin 3-O-glucoside (Mv 3-O-glc).

### Economic assessment

The economic assessment of Mx was carried out by means of the Net Present Value (NPV) approach. The financial tool of NPV has been widely applied in the agricultural sector to decide on the adoption of a project/investment [32, 33]. NPV compares the future cash flow generated by an investment discounted at the baseline year with the adoption cost of the investment. Formally:

$$NPV=-I+\sum_{t=1}^{T}\frac{C_{t}}{{\left(1+r\right)}^{t}}$$

Where, *I* is the cost of the investment, *C*_*t*_ is the net cash flow (revenue minus costs) generated by the investment in the period *t*, *T* is the lifetime of the investment, and *r* is the discount rate. A positive NPV means that the current values of investment-generated future cash flow is higher than the investment cost and thus it is desirable to invest. Conversely, if the NPV is negative the investment should not be made as the future cash flow it generates does not compensate the initial investment cost. We applied the NPV approach to two cases. The first concerns a two hectare vine farm, which is close to the current average vineyard size in Italy. The other deals with the Mossi farm boasting a total of 55 hectares, 30 of which planted with 6 red grape cultivars.

The first step in calculating the NPV is to determine net cash flow annually generated by the investment over its lifetime. The evaluation of Mx assumes that while Mx does not affect (or affects marginally) the revenue of the vine grower, its adoption does influence costs. Indeed, Mx allows cost savings due to its ability to provide field measures of grape components that must be detected during the ripening and just before the harvest, which results in a sharp reduction of labor costs for sampling and it avoids wet chemistry analysis in the laboratory. Thus, the net cash flow in our study represents the difference between the costs the vine grower should afford for the wet chemistry analyses in the laboratory without the Mx and the costs which should be afforded with Mx, i.e. the cost saved if Mx is bought.

We assume that a grapevine cultivar should be sampled 6 times during the season to collect information on the ripening dynamic and each time 5 samples should be taken for each cultivar from different blocks. Hence, the total number of samples is 6 (number of repeated sampling) *5 (number of samples taken each time for each grapevine cultivar) * number of grapevine cultivars. In addition, we assume that the cultivar is sampled one time for the analysis at harvest by taking three bunches per vine and four vines per variety.

The net cash flow (costs without Mx minus the costs with Mx) in our study is composed by different components: (i) costs related to the grape destroyed for the traditional lab analyses (destructive method), (ii) costs related to the labor units for sampling and (iii) costs of lab analyses.

The value of grape destroyed by the destructive method has been computed by considering that grape weight of one sample for ripening analyses ranges between 0.05 and 0.2 kg and the average wholesale grape price ranges between 0.45–0.60 EUR/kg. We also suppose that a bunch very close to harvest weights between 0.1 and 0.4 kg. While this value represents a cost for the destructive method, it is obviously equal to zero for Mx.

Another cost component concerns the labor force employed in the sampling process whose unitary value is around 8 EUR/hour which is the average cost of labor for grape sampling in the area under study. In order to compute the total labor cost for sampling we assume that in a two hectare vineyard the vine grower takes on average 11 minutes for collecting a sample for ripening curves analyses and 4 minutes and a half for collecting a bunch for grape harvest analyses. These values include the time spent to move from one sampling slot to another. The timing of sampling is much faster in the case of Mx as it does not require any grape collection but just moving from one sampling point to another; thus we consider 5 minutes for each plot. This sampling time can be considered the average sampling time for a two-hectare vineyard with a moderate slope. In the case of the larger Mossi vineyard (30 hectares grown with reds), the time for collecting samples for ripening and harvest analyses without Mx was set at 20 and 9 minutes, respectively, while sampling time with Mx was set at 9 minutes and a half. A sensitivity analysis was also run to check the robustness of results under changing sampling time. Finally, the cost of wet chemistry analysis in laboratory for TSS and for ANTH is around 5 EUR/sample and 20 EUR/sample, respectively, and this cost concerns only the destructive method.

The current cost for purchasing Mx is 13,900 EUR and the maintenance cost is around 1,000 EUR every 3 years. Hence, the discounted future net cash flow must be compared with 13,900 EUR plus the discounted maintenance costs according to the lifetime of Mx. The discount rate used is 2%. As technological advances related to analytical equipment are rather quick, we set a lifetime for Mx of seven years.

As we expressed the weight of each grape sample as well as the grape price as a range, we repeated the NPV analysis taking first the lower bound for all the ranges, then their upper bound and finally the average range values. The assessments which take the lower and the upper bound of each range must result, respectively, in the lowest and highest value for the future net cash flow among all possible combinations.

In order to account for the uncertainty related to the cost of labor and to the sampling timing (which depends on many factors such as the way the plots are allocated, the farmland slope, the weather and soil conditions, etc.) we performed Monte Carlo simulations. Monte Carlo simulations allowed us to obtain a distribution for the NPV based on the stochastic behavior of labor cost and sampling timing. We randomly drew from the distributions of the stochastic variables (labor costs and sampling timing) 1000 times and each time the NPV was computed. The simulations were repeated three times: one taking the lower bound of the range for the grape weight and price, one taking the upper bound of each range and one the average.

### Statistical analyses

Data were subjected to a two-way analysis of variance using the SigmaStat software package (Systat Software, San Jose, CA). The cultivar x cluster-side interaction was analyzed only if significant at the F test. Mean separation within cultivars was performed by the Student-Neuman-Keuls test at *p* ≤ 0.05, while t-test was used to separate cluster-side means at the same probability level. Correlation between Mx indices and wet chemistry analytical parameters was tested by regression analyses and all models were calculated using the TableCurve2D Software (Systat Software, San Jose, CA, USA).

## Results

### Weather course, berry growth and ripening

Summer weather in 2017 was characterized by hot conditions, with several hot spells (on 9 days between 1 July and harvest T_max_ exceeded 35 °C) (S1 Fig). These warm conditions led to a Winkler Index summing up to 2143 °C. Total rainfall recorded between 1 June and 30 September was 150 mm and effective rain (49 mm) took place only at the end of July (DOY 209).

**Fig 1:**
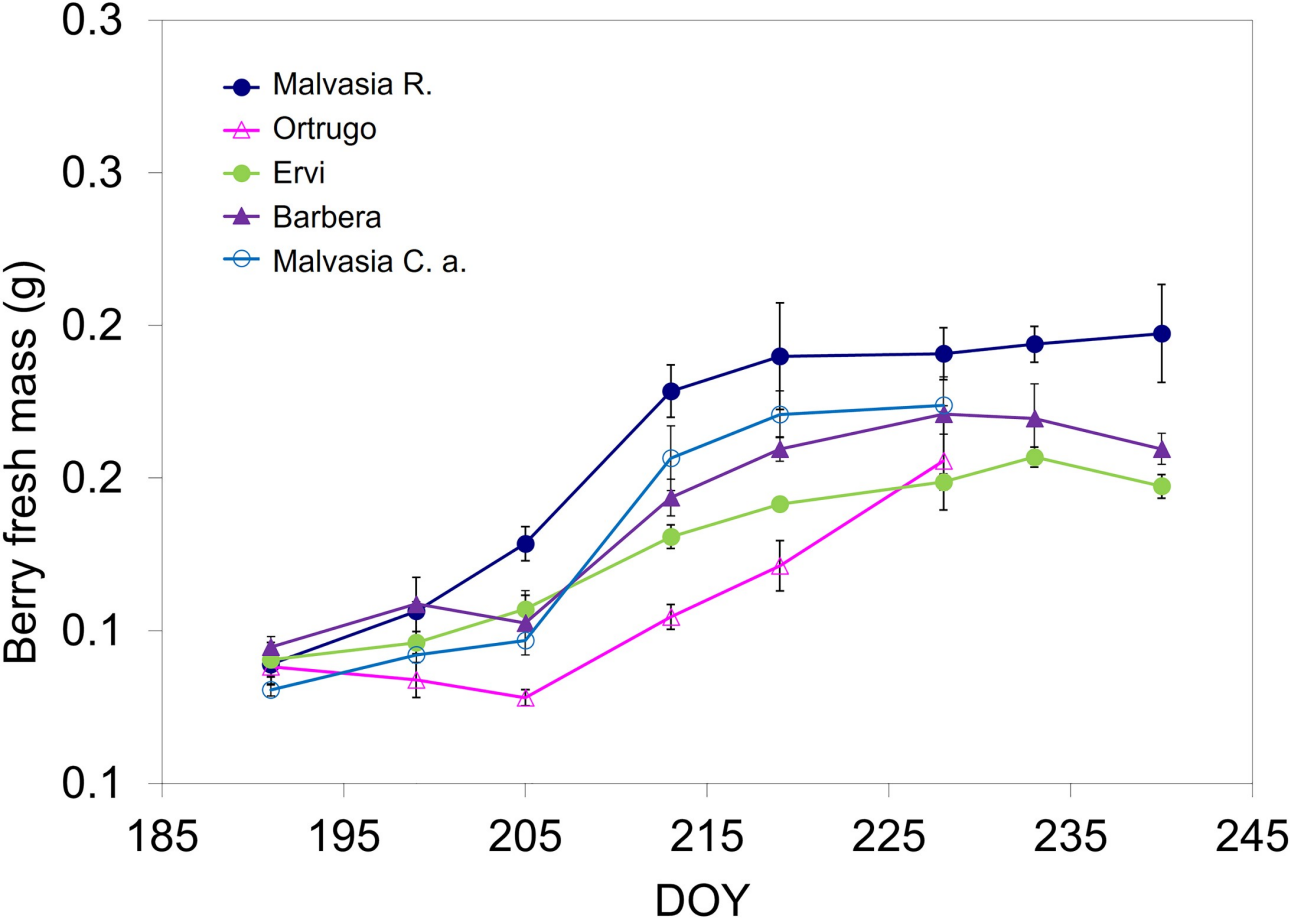
Seasonal progression of berry fresh mass recorded over 8 dates from berry lag-phase to pre-harvest in the 5 cultivars. For each cultivar, within date variation around mean is given by standard error (SE) with n = 3. In a few cases, SE is lower than symbol size.

Berry growth curves for the different varieties showed that until about DOY 205 berries were still in lag-phase and that a linear boost of growth was prompted by the abundant rainfall of DOY 209 (Fig 1). Maximum berry mass was reached in all varieties around mid-August although afterward—especially in Barbera and Ervi—some dehydration occurred. Cluster and berry weight recorded at harvest ranged, across varieties, between a minimum of 170 g and 1.47 g to a maximum of 247 g and 1.95 g (Table 1). The seasonal variation of the skin-to-berry fresh mass ratio (%) is shown, for each cultivar, in S1 Table.

**Table 1:** Cluster weight, berry weight and main must composition parameters recorded at harvest on the five cultivars and, for each of them on the two opposite sides of the same cluster.

	Cluster weight (g)	Berry weight (g)	TSS (°Brix)	TA (g/L)	pH	Tartaric acid (g/L)	Malic acid (g/L)	Total anthocyanins (mg/g)	Total phenols (mg/g)	Total flavonols (mg/g)
***Cultivar***										
Ortrugo	266 a	1.81 b	22.3 b	4.96 c	3.14 a	4.66 b	0.23d	n.d.	1.93 e	1.35b
Malvasia C.a.	247ab	1.69 c	19.9 c	6.38 b	2.95 c	5.51 b	0.57c	n.d.	2.50 c	1.19b
Malvasia R.	274 a	1.95 a	22.9 b	6.04 b	3.17 b	4.83 b	0.86b	0.20 c	2.23 d	0.68a
Barbera	170 b	1.62 c	27.2 a	9.21 a	3.07b	7.47 a	0.88b	1.68 b	2.89 b	2.11c
Ervi	224 ab	1.47 d	26.7 a	6.67 b	3.19a	5.67 b	1.11a	1.91 a	3.78 a	2.84d
*Sig*.	-	**	**	**	**	**	**	**	**	**
***Cluster side***										
North-East Facing	-	1.72	23.63	6.66	3.1	5.7	0.76	0.78	2.63	1.61
South-West facing	-	1.70	23.63	6.64	3.1	5.5	0.70	0.79	2.70	1.67
*Sig*.	-	*ns*	*ns*	*ns*	*ns*	*ns*	*ns*	*ns*	*ns*	*ns*

C x CS interactions were never significant (not shown). Sample size (n) was 24 for the cultivar factor and 60 for the cluster side factor. Data per cultivar are averages over cluster sides. Data per cluster side are averages over cultivars. Mean separation within columns for the cultivar factor by Student Newman Keuls (SNK) test.

** and *ns* stand for significant at p ≤ 0.01 or non-significant, respectively.

**Fig 2:**
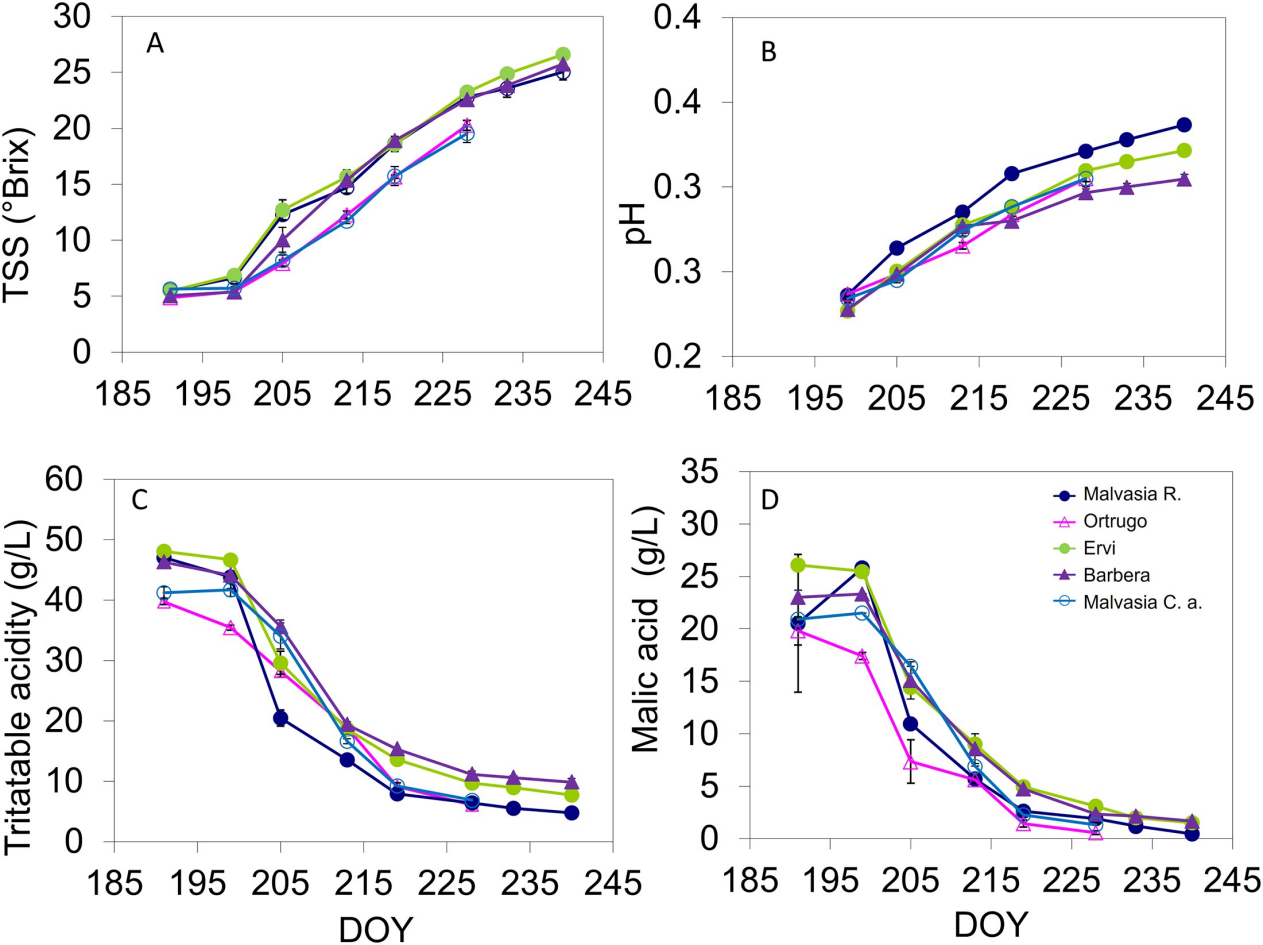
Seasonal progression of total soluble solids (TSS) (A, °Brix), pH (B), titratable acidity (TA) (C, g/L) and malic acid (D, g/L) recorded over 8 dates from berry lag-phase to pre-harvest in the 5 cultivars. For each cultivar, within date variation around mean is given by SE with n = 3. In a few cases, SE is lower than symbol size.

**Fig 3:**
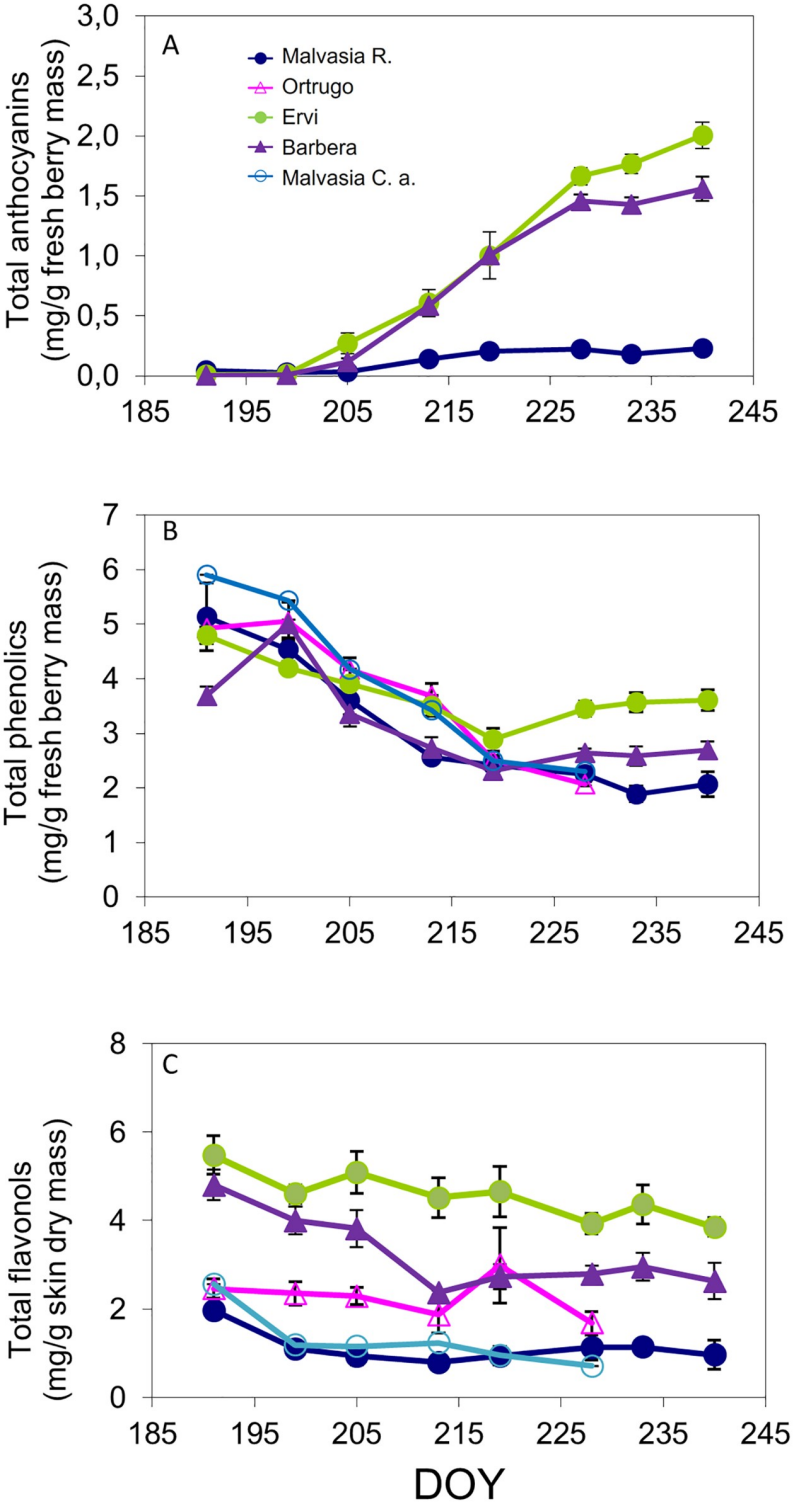
Seasonal progression of total anthocyanins (A, mg/g of fresh berry mass), total phenolics (B, mg/g of fresh berry mass) and total flavonols (C, mg/g of skin dry mass) recorded over 8 dates from berry lag-phase to pre-harvest in the 5 cultivars. For each cultivar, within date variation around mean is given by SE with n = 3. In a few cases, SE is lower than symbol size.

The berry sugaring process occurred smoothly in all varieties (Fig 2A), at harvest reaching around 20 °Brix in Malvasia C.a., whereas Ortrugo progressed to reach a TSS level (22.3 °Brix) quite similar to that recorded in Malvasia R. (Table 1). Ervi and Barbera set at harvest around the unseasonally high 27 °Brix as a likely result of some berry water loss. Seasonal pH curves showed that Malvasia R. tended to maintain higher pH throughout the ripening season, although at harvest its final must pH was not different from that of Ortrugo and Ervi, whereas Malvasia C.a. and Barbera were the lowest (Fig 2B). As per total titratable acidity (TA), Ortrugo was the variety having the lowest pool of acids on DOY 190 (Fig 2C) and likewise the lowest level at harvest (4.96 g/L) (Table 1). Remarkably, despite a final TSS of 27.2 °Brix, Barbera was able to retain a final TA of 9.21 g/L. Malic acid degradation was especially fast in Ortrugo (Fig 2D) and very little malate (0.21 g/L) was retained in Ortrugo’s berries at harvest. In general, malic acid concentration at harvest was very low in all cultivars, with Ervi standing out at 1.11 g/L. Progression of total anthocyanins accumulation and final level reached at harvest indicated Ervi as the most efficient for berry pigmentation (1.91 mg/kg) (Fig 3A). Ervi also showed the highest total phenolics over the last part of the season (Fig 3B) and a progressively decreasing total phenols concentration was shown at harvest in the following order: Ervi > Barbera > Malvasia C.a. > Malvasia R. > Ortrugo (Table 1). In terms of single anthocyanidin composition determined at harvest by HPLC analysis, 97% of total anthocyanins in Malvasia R. was accounted by cyanidin and peonidin, while in Ervi and Barbera malvidin was largely the most abundant compound followed by petunidin (not shown). Ervi again displayed the highest total flavonols concentration (mg/g dry skin mass) during the ripening season (Fig 3C), whereas Malvasia R. and Malvasia C.a. had quite low levels. Such differences were confirmed at harvest, with Ervi scoring maximum flavonols concentration (2.84 mg/g) and Malvasia R. the lowest (0.68 mg/g).

For none of the measured must composition parameters significant differences were found at harvest when the two opposite South-West facing and North-East facing side of the cluster were compared (Table 1). Analysis carried out on absolute and relative growth of single berry components (i.e. skin, flesh and seeds) showed that, when given on a relative basis, between-cultivar differences were quite mild (Table 2). Notably, skin-to-flesh and skin-to-berry ratios were higher on Barbera as compared to the other cultivars.

**Table 2:** Absolute and relative growth of berry components (skin, seeds and flesh) and number of seeds per berry recorded at harvest on the five cultivars.

	Skin weight (g)	Total Seed weight (g)	Flesh weight (g)	Seeds/Berry (n)	Single seed weight (mg)	Skin-to-berry ratio (%)	Total seed-to-berry ratio (%)	Flesh-to-berry ratio (%)	Skin-to-flesh ratio (%)
Ortrugo	0.155b	0.0599 b	1.515c	1.56b	38.26c	8.96a	3.46a	87.58b	10.23a
Malvasia C.a.	0.151b	0.0582b	1.527c	2.15d	28,33a	8.70a	3.35a	87.95b	9.89a
Malvasia R.	0.172b	0.0747d	1.648d	2.75e	27.71a	9.08a	3.94b	86.98ab	10.44a
Barbera	0.159b	0.0674c	1.392b	1.96c	34.51b	9.82b	4.16b	86.01a	11.42b
Ervi	0.135a	0.0474a	1.261a	1.37a	34.90b	9.35a	3.29a	87.36b	10.71a
*Sig*.	**	**	**	**	**	*	*	*	*

For each cultivar, 240 berries were individually processed. Mean separation within columns for the cultivar factor by Student Newman Keuls (SNK) test.

** and * stand for significant at p ≤ 0.05 and 0.01, respectively.

### Field Mx vs. wet chemistry

**Fig 4:**
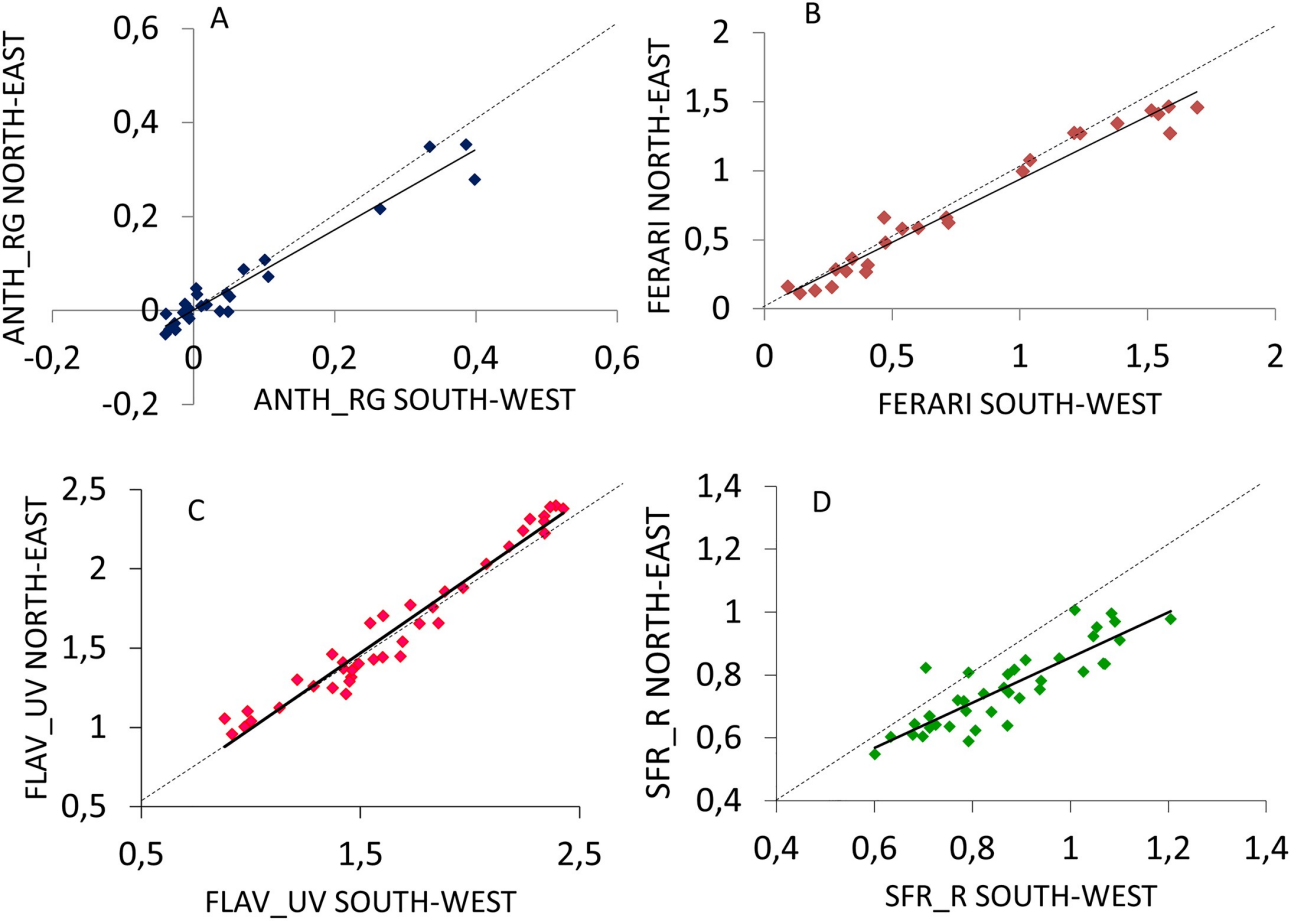
Correlations between field recorded Mx readings taken during the season on the two opposite sides of the same cluster (South-West and North-East facing) and analyzed for ANTH_RG (A), FERARI (B), FLAV-UV (C) and SFR_R (D). In each panel, dots represent dates x cultivar combinations. Within dates and cultivars, data were pooled over cluster side and cluster and vine replicates (n = 24). Within each panel, data were pooled over sampling dates and cultivars. All data shown in panels A-D were fitted to the following linear models: y = 0.8537x + 0.0014, R^2^ = 0.94 (A); y = 0.9131x + 0.0026, R^2^ = 0.97 (B); y = 0.951x + 0.0433, R^2^ = 0.95 (C); y = 0.7180x + 0.1372, R^2^ = 0.75 (H). Sample size (n) is 27 in panels A and B (cultivars Malvasia R., Ervi and Barbera), and 41 in panel C and D (all cultivars). In each panel, the dotted line indicates the 1:1 relationship.

Seasonal variation of field Mx measurements of SFR_R (A), FLAV_UV (B) and FERARI (C) in the five cultivars is shown in S2 Fig. For data pooled over cultivars and sampling dates, a tight linear correlation (R^2^ = 0.999) was found between FLAV_UV calculated according to [16] and FLAV_UV calculated as the sun of FLAV and FERARI (S3 Fig). A very close linear correlation (R^2^ > 0.94) occurred between both ANTH_RG and FERARI indices for field Mx measurements taken on the two opposite sides of the same cluster (Fig 4A and 4B). FLAV-UV and SFR_R also showed a tight linear correlation between Mx readings taken on the two opposite cluster sides (Fig 4C), whereas the linear model calculated for SFR_R explained 75% of total variability (Fig 4D). Notably, the latter case showed that at fairly high SFR_R values (hence earlier in the ripening season) the South-West exposed side of the cluster prompted a slightly advance sugar accumulation that, however, offset with progressing ripening.

**Fig 5:**
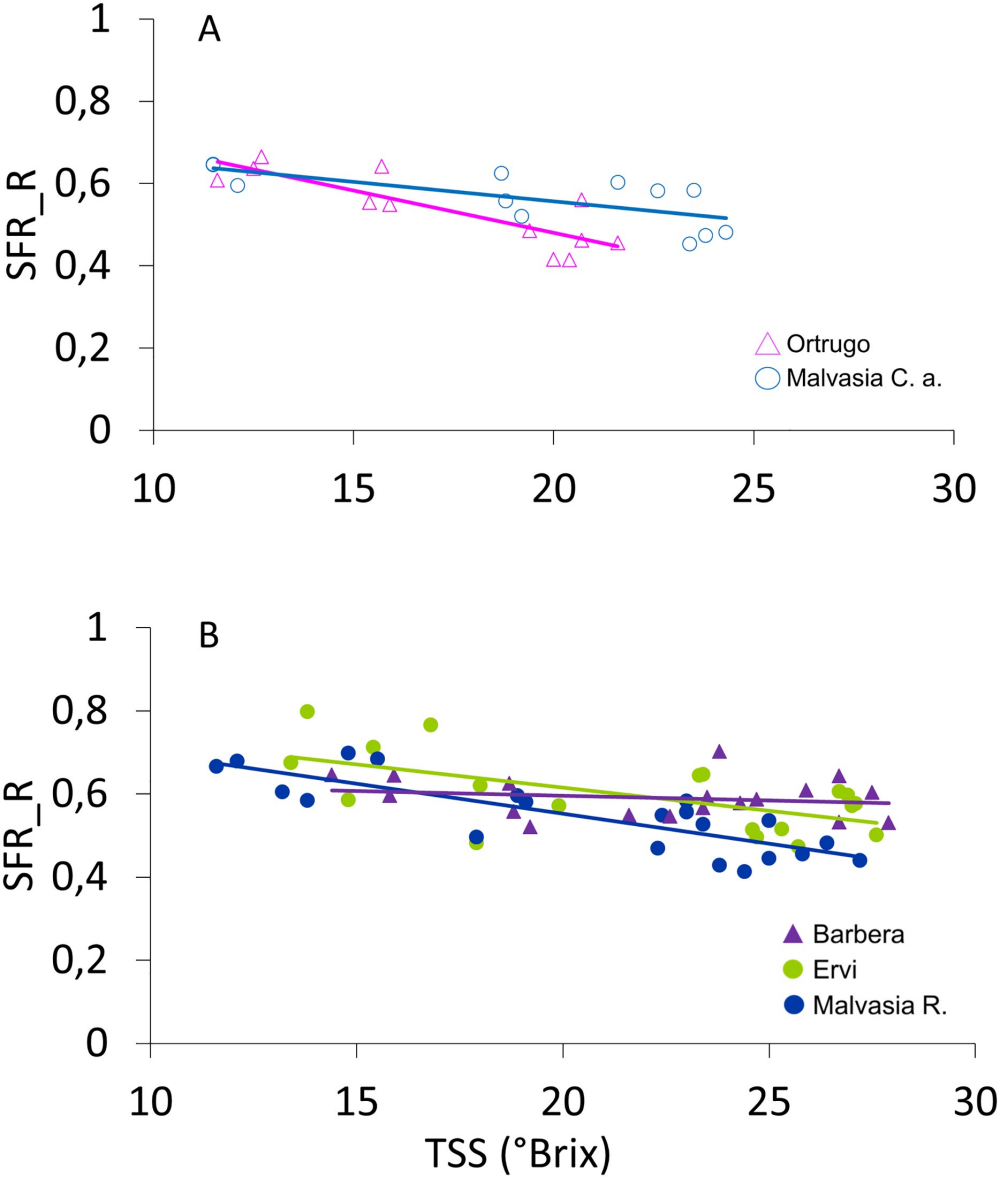
Correlation between field recorded SFR_R index and TSS (°Brix) in the five cultivars grouped as white (A) and colored (B), for TSS values ≥ 10° Brix. In all cases except for Barbera, data were fitted to a significant negative linear model (P < 0.05) having the following equations: y = -0.0205 + 0.8906, R^2^ = 0.71, n = 12 (Ortrugo): y = -0.0096 + 0.7476, R^2^ = 0.50, n = 12 (Malvasia C.a.); y = -0-0144x + 0.8405, R^2^ = 0.68, n = 21 (Malvasia R.); y = -0.063x + 0.7375, R^2^ = 0.04, n = 21 (Barbera); y = -0.0112x + 0.8398, R^2^ = 0.37, n = 21 (Ervi).

When total soluble solids (TSS) determined by refractometer analysis were preliminarily regressed over field-based Mx readings with data pooled over the five cultivars (S4 Fig), data distribution identified a cloud of data including TSS ranging between 4.5 and 9.2 °Brix comprised within a highly variable interval of the SFR_R index (0.5–1.1 Mx units) and a second data group collecting TSS ≥ 10 °Brix, a threshold beyond which, a more consistent negative linear relationship between SFR_R and wet-chemistry TSS was shown. More precisely, R^2^ values of linear correlations drawn for each of the five tested cultivars varied from a maximum of 0.71 in Ortrugo to a minimum of 0.37 in Ervi, while no correlation was found in Barbera (Fig 5).

**Fig 6:**
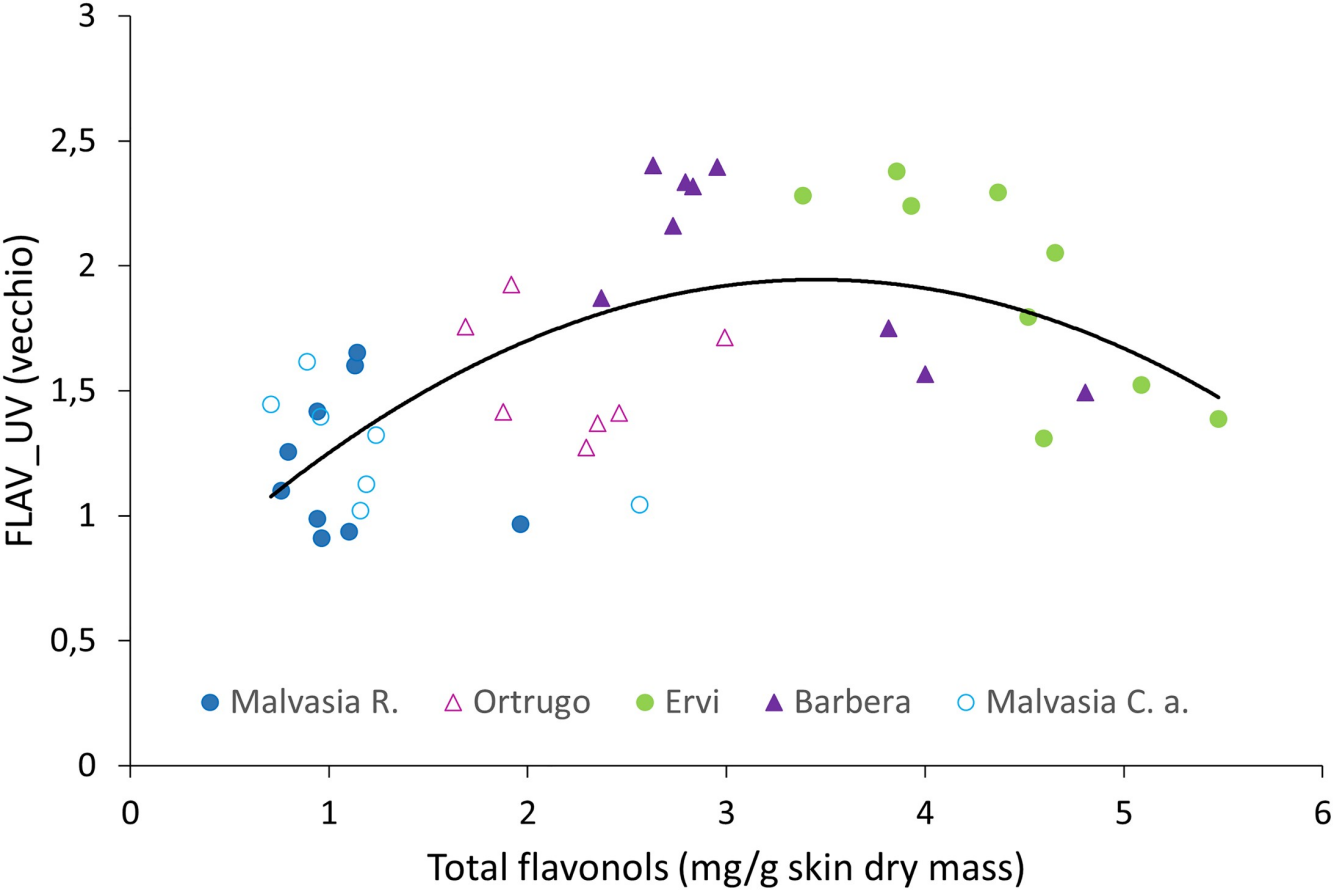
Correlation between field recorded FLAV_UV and total flavonols (mg/g dry skin mass) for data pooled over the five cultivars (n = 41). Data were fitted to the following curvilinear model: y = -0.1105x^2^ + 0.7613x + 0.628, R^2^ = 0.35 (P = 0.05). Closest fits were reached for cv. Barbera (y = -0.1331x2 + 0.5898x + 1.589, R^2^ = 0.66, P < 0.01, n = 9) and Ervi (-0.0886x2 + 0.2592x + 2.5412, R^2^ = 0.64, P < 0.01, n = 9).

For data pooled over cultivars and dates, no correlation was found between FLAV and total flavonols concentration determined by HPLC (S5 Fig). However, when the FLAV_UV index was regressed with the total flavonols concentration a significant curvilinear model was calculated for Barbera (y = -0.0036x + 3.1891, R^2^ = 0.63) and Ervi (y = -0.0053x + 4.2462, R^2^ = 0.64), while no correlation was confirmed for the two white cultivars and for Malvasia R. (Fig 6).

**Fig 7:**
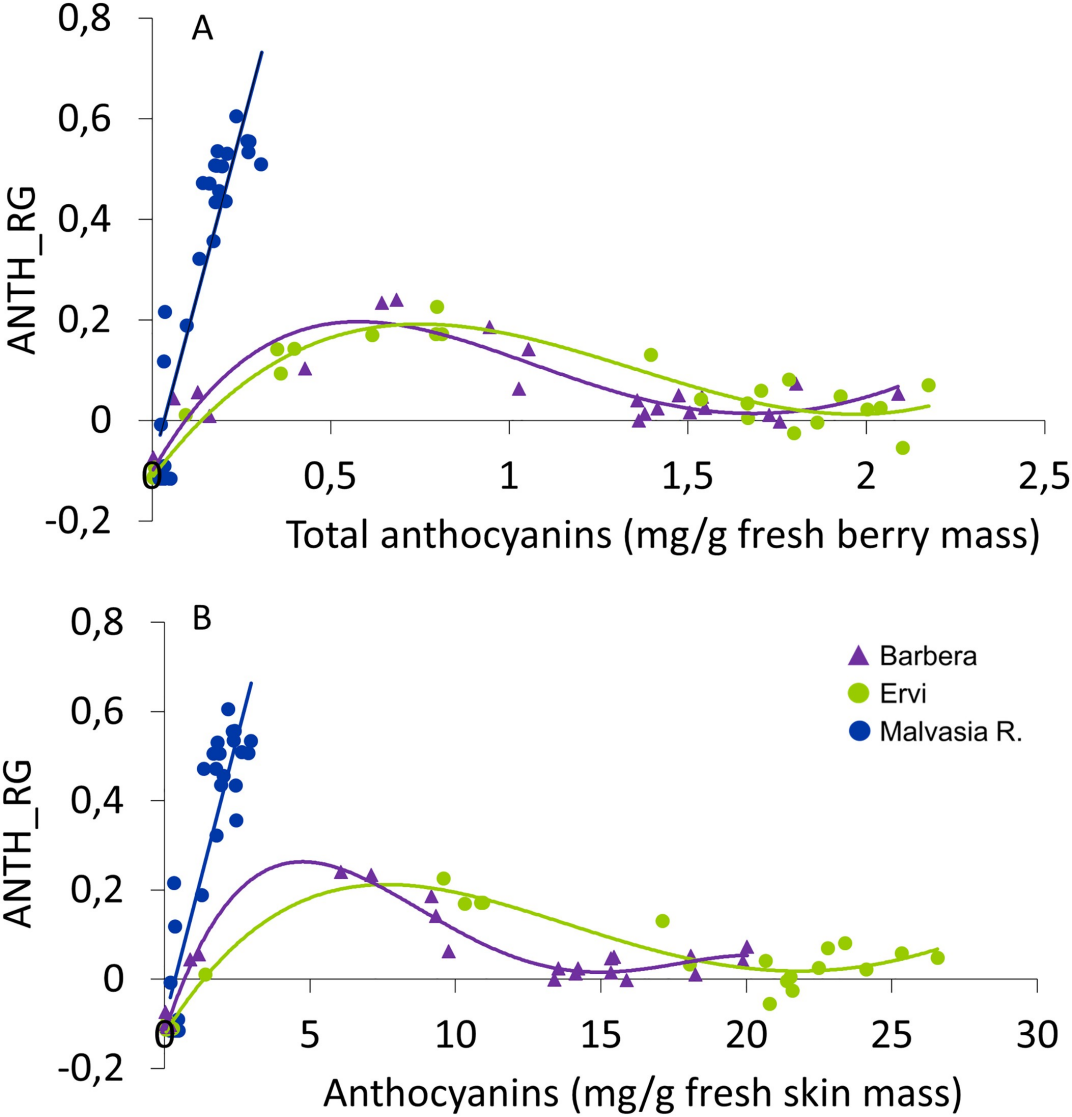
Correlation between seasonal field recorded ANTH_RG index and the ANTH concentrations determined by wet chemistry in three red cultivars with ANTH given on a berry mass fresh basis (A) and a skin mass fresh basis (B). In panel A, data taken on the low color-accumulating Malvasia R. fitted to the following linear model: y = 2.6675x − 0.0891, R^2^ = 0.82., n = 27. Data taken on Barbera and ERVI were instead fitted to the following polynomial models: y = -0.102x^4^ + 0.7315x^3^- 1.6365x^2^–1.2373x - 0.102, R^2^ = 0.85, n = 27 (Barbera) and y = -0.0081x^4^ + 0.2422x^3^–0.8925x^2^ + 0.9441x − 0.1142, R^2^ = 0.91, n = 27. In panel B, Malvasia R. data were fitted to the following linear model: y = 0.2527x − 0.0893, R^2^ = 0.79, n = 27. Polynomial models run through Barbera and Ervi data were: y = -3E-05x^4^ + 0.0018x^3^- 0.0319x^2^ + 0.1946x − 0.1191, R^2^ = 0.94, n = 27 (Barbera) and y = -4E-06x^4^ + 0.0004x^3^- 0.0106x^2^+0.1057x -0.1238, R^2^ = 0.91, n = 27.

Irrespective of their expression unit (per total berry mass or per skin fresh mass), total anthocyanins were closely and linearly correlated with ANTH_RG in Malvasia R. In the two red cultivars, a polynomial model was fitted to the data showing that a peak in the ANTH_RG index was reached for a total anthocyanin concentration comprised between 0.5–1.0 mg/g and, afterward, ANTH_RG gradually declined to approach close to zero values between 1.5 and 2.2 mg/g (Fig 7A). Running the same correlation with total anthocyanins on a per skin mass basis did not change the model shape, although the model precision considerably improved in Barbera (R^2^ = 0.94) while no enhanced precision was shown in Ervi (R^2^ = 0.91, regardless of unit of data expression) (Fig 7B).

**Fig 8:**
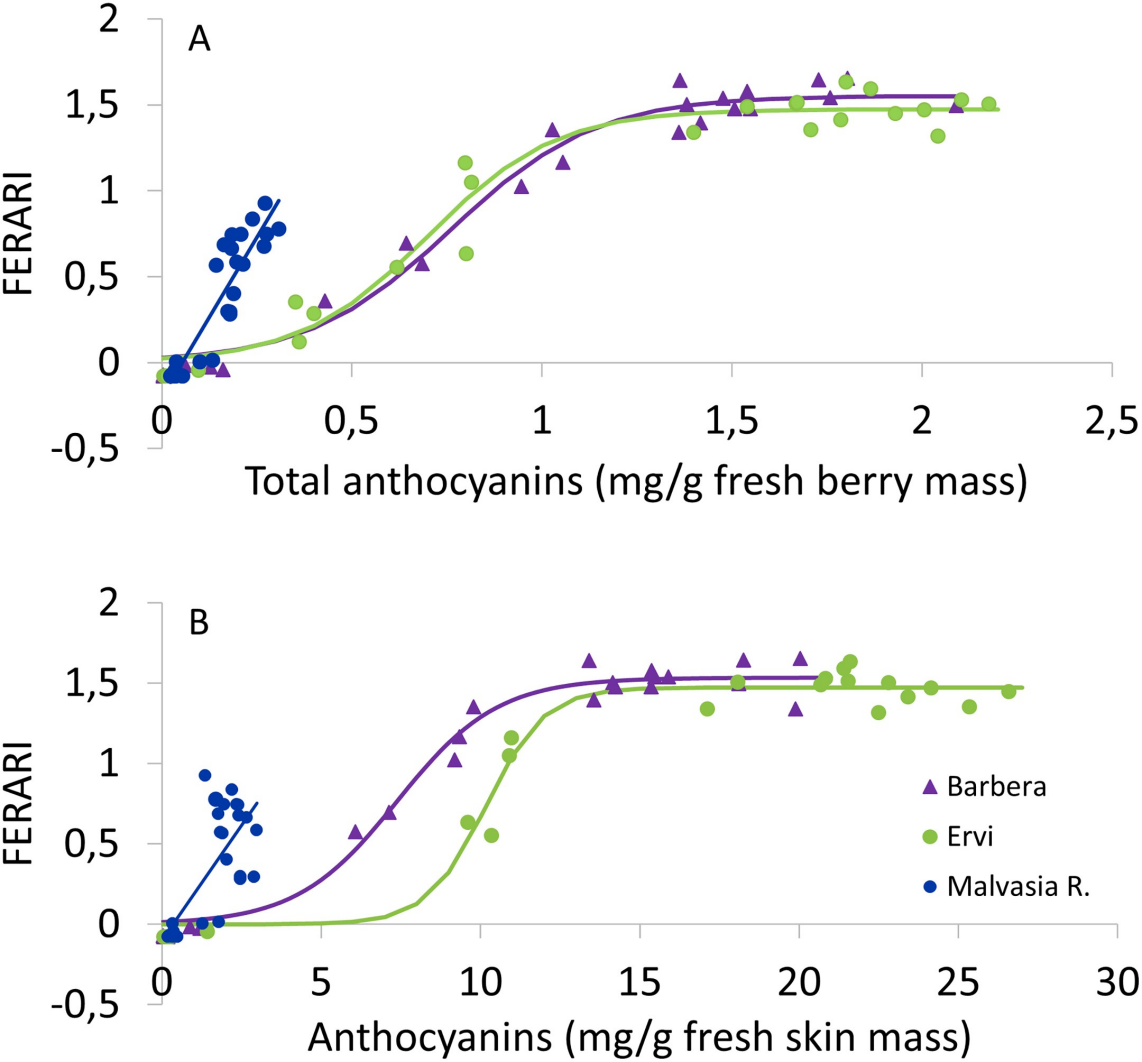
Correlation between seasonal field recorded FERARI index and the Anth concentrations determined by wet chemistry in three red cultivars with Anth given on a berry fresh basis (A) and a skin fresh basis (B). In panel A, data taken on the low color-accumulating Malvasia R. fitted to the following linear model: y = 3.7167X − 0.1982, R^2^ = 0.84. Data taken on Barbera and Ervi were instead fitted to the following sigmoid models: y = 1.55/(1+EXP(4.005–5.261x)), R^2^ = 0.99, n = 27 (Barbera) and y = 1.47/(1+EXP(4.132–5.906x)), R^2^ = 0.97, n = 27 (Ervi). In panel B, Malvasia R. data were fitted to the following linear model: y = 0.313x − 0.1403, R^2^ = 0.64, n = 27. Sigmoid models run through Barbera and Ervi data sets were: y = 1.533/(1+EXP(4.690–0.635x)), R^2^ = 0.99, n = 27 (Barbera) and y = 1.471/(1+EXP(11.064–1.088x)), R^2^ = 0.98, n = 27.

Correlating FERARI index calculated from field readings with total anthocyanins concentration determined in Malvasia R. berries showed that precision of the linear model lost some accuracy when data were given on a per skin weight basis (R^2^ = 0.64) against R^2^ of 0.84 scored for data expressed on a whole berry mass basis. A very close correlation was found between FERARI index and total anthocyanin concentration recorded in the two red cultivars, regardless of the unit chosen to express the data (Fig 8). The sigmoid model fitted to the data showed a plateau threshold above 1.5 mg/g of fresh mass. Interestingly, expressing data on a per skin mass basis allowed a better identification of the plateau threshold that set at about 15 mg/g in Barbera and at 20 mg/g in Ervi.

### Economic section

The results of this study show that while Mx provides a precise measure of ANTH concentration in grape, the detected TSS measure is not always reliable. Thus, the cost saving of Mx concerns only the ANTH, while the winegrower must gather information on TSS from other tools. As most winegrowers own a refractometer which provides a precise measure of TSS, we assume that TSS is detected by a refractometer and thus no lab analyses are required if Mx is bought. Therefore, the economic assessment of Mx is focused only on grapevine red varieties due to the most important feature of Mx, i.e. its ability to detect precisely the grape ANTH concentration.

We assessed the economic suitability of purchasing Mx for a two-hectare vineyard and for the Mossi vineyard (30 hectares planted with red varieties). Notably, in none of the two cases there was significant difference in the expected value for NPV comparing the three trials: lower bound, upper bound and average level for the grape price and grape weight ranges (Table 3). Assuming a lifetime for Mx of seven years, the expected value of NPV in the case the two-hectare farm grows 3 red cultivars (corresponding to 15 samples per date) is positive and statistically different from zero (-1,169 EUR) indicating that the costs of buying and maintaining Mx are more than compensated by the future seven-year saved costs (Table 3). Seven years are also the minimum number of years required to reach a positive expected NPV for this vine farm cultivating 3 red cultivars. Monte Carlo simulations on the same sized vineyard cultivating only two red varieties results in a negative and statistically significant expectation for NPV. In this case the vine grower should use Mx for at least 11 years to make the investment desirable. Results from the Mossi vine estate indicate an economic convenience for the estate to invest in Mx as the expected NPV with a lifetime for Mx of 7 years is largely positive and significant. Mossi vineyard should use Mx for at least three years to make the Mx purchase desirable, which is a time horizon much shorter than the expected lifetime of the instrument. The sensitivity analysis shows that increasing by the same percentage the sampling time with and without Mx leads to no change in the results. In addition, when the sampling time from Mx is set equal to the sampling time without Mx (that is a rather unreasonable hypothesis and can be considered an upper bound for the sampling time with Mx) results are confirmed up to a sampling time value of 30 minutes.

**Table 3:** Expected NPV and minimum Mx lifetime according to the number of grapevine red varieties grown in the vineyard.

	Lower range bound	Upper range bound	Range average
**Two-hectare vineyard**			
*Two cultivars*			
NPV (euro) if the Mx lifetime is 7 years	-4,487***	-4,435***	-4,463***
Minimum number of years to have a positive NPV	11	11	11
*Three cultivars*			
NPV (euro) if the Mx lifetime is 7 years	1,135***	1,212***	1,169***
Minimum number of years to have a positive NPV	7	7	7
**Mossi vineyard (30 hectares as red cultivars)**			
NPV (euro) if the Mx lifetime is 7 years	18,930***	19,086***	19,000***
Minimum number of years to have a positive NPV	3	3	3

The range refers to the ranges of grape sample weight for ripening analyses (50–200 grams), of grape bunch weight for harvest analyses (100–400 grams) and of the grape price (0.45–0.60 euro/kg). The lower bound takes the lowest values of each range, the upper bound takes the highest values of each range and the average takes the average value of each range.

*** indicates a P < 0.001 statistical significance.

## Discussion

First goal of this work was to assess if, for a range of technological and phenolic ripeness parameters assessed on a number of either white and red cultivars grown in the Colli Piacentini wine district, Mx indices taken in the field could provide reliable enough to effectively replace the quite time-consuming process of destructive berry sampling in field. Albeit limited to a single season, variability assured by the range of chosen genotypes and their interaction with a quite hot season with limited rainfall in summer was undeniable: TSS ranged, at harvest, from 19.9 °Brix (Malvasia C.a.) to 27.2 °Brix (Barbera); TA went from the quite low concentration of 4.96 g/L in Ortrugo to the peak of 9.21 g/L scored in Barbera, the highest total phenolics concentration reached in Ervi (3.78 mg/g) almost doubled that measured in Ortrugo (1.93 mg/g) while total ANTH varied from the very low 0.20 mg/g in the pink colored Malvasia R. to the notable 1.91 mg/g in Ervi. Despite increasingly criticized, TSS is still the most widely considered ripening parameter in viticulture simply because final price tags are often built on reaching a given TSS threshold. Overall, in our study, TSS estimates obtained through the SFR_R index taken in the field with Mx were not accurate enough to validate its use in replacement of traditional wet chemistry or other approaches, regardless of correlation being run within single cultivars or data pooled over cultivars. Such response does not appear to fit with previous work done by [12, 15, 16] reporting correlations having R^2^ > 0.80. In our trial, the white cv. Ortrugo showed the closest fit with (R^2^ = 0.71) (Fig 5A). Another clear outcome of our study was that, as long as the TSS stays between the 4 to 10 °Brix range, SFR_R is not a good proxy for its estimation. In physiological terms, this is not too surprising. If the SFR_R essentially works as a reliable estimator of the rate of chlorophyll degradation during a ripening or senescence process, it is very well known that the initial rapid surge of sugar up until 8–9 °Brix in the grape berry takes places during the softening phase when the berry becomes translucent without any change in pigmentation [34]. It can therefore be speculated that during the initial sugaring process (roughly 4 to 8–9 Brix) there is a change in the berry structure that modifies the scattering properties of tissues increasing more the FRF_R signal with respect to the RF_R signal, independently of the chlorophyll content. Color appearance and, hence, chlorophyll degradation, starts to show up at TSS > 9–10 °Brix that is exactly the threshold beyond which the SFR_R became more precise in predicting actual concentration.

About precision of the FLAV index in estimating total flavonols concentration, our data is in agreement with that reported by [16] in Barbera grapes and by [35] in Aleatico grapes showing very poor correlation that, in our study, applied to all cultivars tested. [16] suggested that such poor correlation is due to the large change in the FRF_R signal induced by the anthocyanins accumulation that, still, does not explain poor performance observed also in our two white cultivars. The same authors reported that the correlation significantly increased in accuracy (R^2^ = 0.74), at least in some cultivars, when the FLAV index was replaced by the FLAV_UV calculated as log(1/FRF_UV). However, calculation of this revised index led to quite contrasting results. From one side, correlation vs. total flavonols determined by wet chemistry greatly improved in the two red cultivars, therefore supporting conclusions previously drawn by [16] for Barbera. On the other side, no substantial improvement was seen in the remaining varieties when FLAV_UV replaced FLAV. The latter outcome fits with the hypothesis of [16] who suggested that the calibration curve between FLAV_UV and total flavonols tend to loose accuracy when data refers to cultivars (in their case Arneis and Timorasso) which show high concentration of flavonols in berries. They also speculated that a saturation threshold of the optical method might occur when the berry flavonols concentration is higher than 150–180 mg/kg. Though, this does not seem to apply to our white cultivars, whose maximum flavonols concentration stayed below such limits. Rather, another factor that might have played a significant role in poor correlation is that almost all flavonols readings were taken between veraison and full ripening. It is well known from the literature [1, 36]. that the seasonal pattern of flavonols accumulation into grape berries is almost linear and fast from fruit-set until veraison and it stays constant or slightly decreases from veraison onward [36]. This has indeed contributed to narrow the variability range of flavonols concentrations and, with it, accuracy of predictions.

While the ANTH_RG index showed a very close linear correlation with the small amount of color that the “pink” Malvasia R. was able to accumulate in berry skin (up to about 0.2 mg/g berry fresh mass), the shape of the correlation shown for data pertaining to Barbera and Ervi (both to be considered as two good color “accumulators”) is a polynomial model showing that ANTH_RG peaks around full cluster coloring (roughly 0.5–0.7 mg/g) and then a reverse phase starts with a linear decline until harvest. The phenomenon has been explained by the fact that ANTH_RG is given by the difference of two components, that is logFRF_R and logFRF_G, that decrease exponentially with increasing color and the component in the green decreases faster than that in the red, due to the larger absorbance of anthocyanins at green wavelengths. Such bi-phasic patterns generate two main limitations: a quite complex calibration of the index that in several instances has been suggested to be overcome by just using the index for post-veraison stages [13] and a quite inherent and apparently unavoidable difficulty if the goal is, for example, estimating the onset of veraison and evaluating its degree of coupling with the sugaring process. However, for the estimation of the onset of veraison other models have been proposed in previous work [12].

[17] have also proposed another challenging hypothesis; that is, part of between-season variability of the relationship between total Anth concentration determined destructively and the ANTH-RG index is due to the unit used to express Anth concentration that is usually based on total berry mass, while it is well known that color accumulates almost entirely in skin tissues. Although we do not have multi-season data in this work, we do have a precise assessment of the relative skin mass (S1 Table) and so we could re-run the regressions using Anth concentration given as mg/g of skin fresh mass. While such a change did not affect shape and accuracy of predictions in Ervi, it increased quite significantly the precision model in Barbera but, most importantly, it disclosed that, for instance, at a given value of the index (e.g. 0.2) Barbera had accumulated much less ANTH than Ervi. Once confirmed by more studies where Mx readings will be coupled with objective assessment of relative skin mass, this would likely achieve a better definition of those cases where using a single calibration model for different cultivars is not suitable.

The FERARI index confirmed its reliability as a very good predictor of Anth accumulation (Fig 8). The very close curvilinear model fitted to Barbera and Ervi data is mono-phasic and very usable. Very interestingly though, and with greater evidence as compared to the previously discussed ANTH_RG, when FERARI was also regressed over anthocyanin concentration given on a skin-mass basis, the curves of the two cultivars markedly shifted apart and, once again, for any index value comprised between 0 and 1.2 Mx unit), while the amount of “estimated color” was very similar between cultivars when data were given on a whole berry, such difference significantly widened when unit of expression was changed to a skin basis. This is very interesting as this alternative color unit allows discriminating cultivars in terms of initial speed of color accumulation, a key factor within a scenario of climate change.

The very high correlation found between values taken, almost simultaneously, on the two opposite sides of the same cluster (South-West facing and North-East facing) for FLAV-UV, ANTH_RG and FERARI indices suggests, on one side, very little incidence of daytime and diffuse-to-direct light ratio on readings and, on the other, that diurnal variation in light hitting the two cluster sides due to row orientation was too mild to affect color and flavonols uniformity within the same cluster as also confirmed by harvest wet chemistry (Table 1). Somewhat higher deviation from the 1:1 line was shown for SFR_R measured on both cluster sides (R^2^ = 0.75) especially during early ripening when the SW exposed side tended to show higher SFR-R, hence slightly slower sugar accumulation rate that the NE exposed part. Hypothesis that can be made is that being local sugar concentration within a single cluster also a function of photosynthetic capacity of the surrounding or adjacent leaves, higher light and thermal exposure pertinent to the SW side might have caused limitation in leaf photosynthesis as well increasing the respiration activity (Table 1). However, it has to be pointed out that between cluster side SRF-R difference narrowed with advancement of ripening and at harvest it was almost nil to confirm wet chemistry data shown in Table 1.

As the strength of Mx concerns ANTH concentration, our economic assessment is focused only on grapevine red cultivars. According to our results, setting a lifetime for Mx of 7 years makes Mx economically attractive if at least 90 sampling are carried out each year. This is easily reachable for larger vine farm (such as Mossi estate) which usually grows a large number of varieties, while it may represent a negative constraint for smaller farms cultivating only one or two red varieties. Although we performed Monte Carlo simulations and a sensitivity analysis to account for the high heterogeneity in the sampling time according to human and natural conditions, our results can be generalized only to vineyard conditions similar in size, slope and weather conditions to the 2 hectare vineyard and to the Mossi vineyard analyzed. Indeed, our conclusions result to be robust within an increase in the sampling time up to 30 minutes/sample. Vineyards that largely differ in slope and weather conditions as compared to the ones analyzed in this study require a rerun of the Monte Carlo simulations.

## Conclusions

Considering five cultivars (two reds, two whites and one pink colored) having different kinetics and accumulation patterns of TSS, total ANTH and flavonols generated, as it was hoped, a quite heterogeneous compositional scenario that confirmed Mx as very efficient and reliable tool for indirect, fast and non-destructive estimates of total ANTH in the ripening berries. This evidence is highly significant and reliable when FERARI index is considered. The same precision was not found for FLAV-UV showing a linear correlation for the two red cultivars only whereas, for TSS, it resulted quite variable with best accuracy reached in cv. Ortrugo in the mid-veraison to harvest interval. Novelty of our work is also that Mx readings were paralleled with concurrent wet chemistry analyses and determination of the weight of single berry organs; thus, correlation of FERARI and ANTH_RG vs. ANTH given on a per skin fresh mass basis could be performed revealing that, while precision of the models was slightly improved, behavior of single cultivars (i.e. maximum amount of accumulated color at a given index value and speed of color accumulation) could be characterized with higher sensitivity. Finally, the economic assessment of Mx carried out through the Net Present Value approach showed that Mx is economic suitable for a 2 hectare vineyard cultivating at least three red varieties (corresponding to 90 samples per year). If the varieties grown are only two, then Mx becomes economic attractive if it is used for at least 11 years. This sheds light on the potential role of cooperatives and producers associations in purchasing collectively Mx and in renting them to their members. On the other side, large vine estates are economically motivated to purchase Mx also individually as they reach a positive NPV from the investment after a few years of utilization.

## Supporting information

S1 Fig Daily weather course as mean (green), maximum (red) and minimum (blue) diurnal air temperature (T) rainfall (mm, vertical bars) and global solar radiation (W/m^2^, broken line) recorded from pre-veraison (DOY 185) until harvest of the two red cultivars (DOY 249) recorded by a weather station located nearby the experimental plots. Arrows indicate harvest dates for Malvasia C.a., Malvasia R., Ortrugo (broken) and Barbera and Ervi (solid).
(TIF)

S2 Fig Seasonal variation of field Mx measurements of SFR_R (A), FLAV_UV (B) and FERARI (C) in the five cultivars. Within each date, Mx readings were pooled over cluster side as well as cluster and vine replicates (n = 24).
(TIF)

S3 Fig Linear correlation between FLAV_UV calculated according to Ferrandino et al 2017 [16] and FLAV_UC calculated as the sum of FERARI and FLAV. Equation of the linear model is: y = 1.0015x -0.6066. Data were pooled over cultivars and sampling dates.
(TIF)

S4 Fig Scatter diagram of field Mx readings of SFR_R and TSS measured in the laboratory through wet chemistry. Data were pooled over sampling dates and cultivars. A clear inflexion point is visible across the 10 °Brix threshold.
(TIF)

S5 Fig Scatter diagram of field Mx readings of FLAV and flavonols concentration measured in the laboratory through wet chemistry. Data represent all sampling dates x cultivar combinations.
(TIF)

S1 Table Evolution of skin to berry ratio (%) across the ripening process on the five cultivars evaluated. (DOCX)

S1 Data Minimal data set. (DOCX)
