# Supplementary figures and images for: Destructive and optical non-destructive grape ripening assessment: Agronomic comparison and cost-benefit analysis
Source: PLoS One. 2019 May 29;14(5):e0216421. doi: 10.1371/journal.pone.0216421 (PMC6541254; doi:10.1371/journal.pone.0216421)

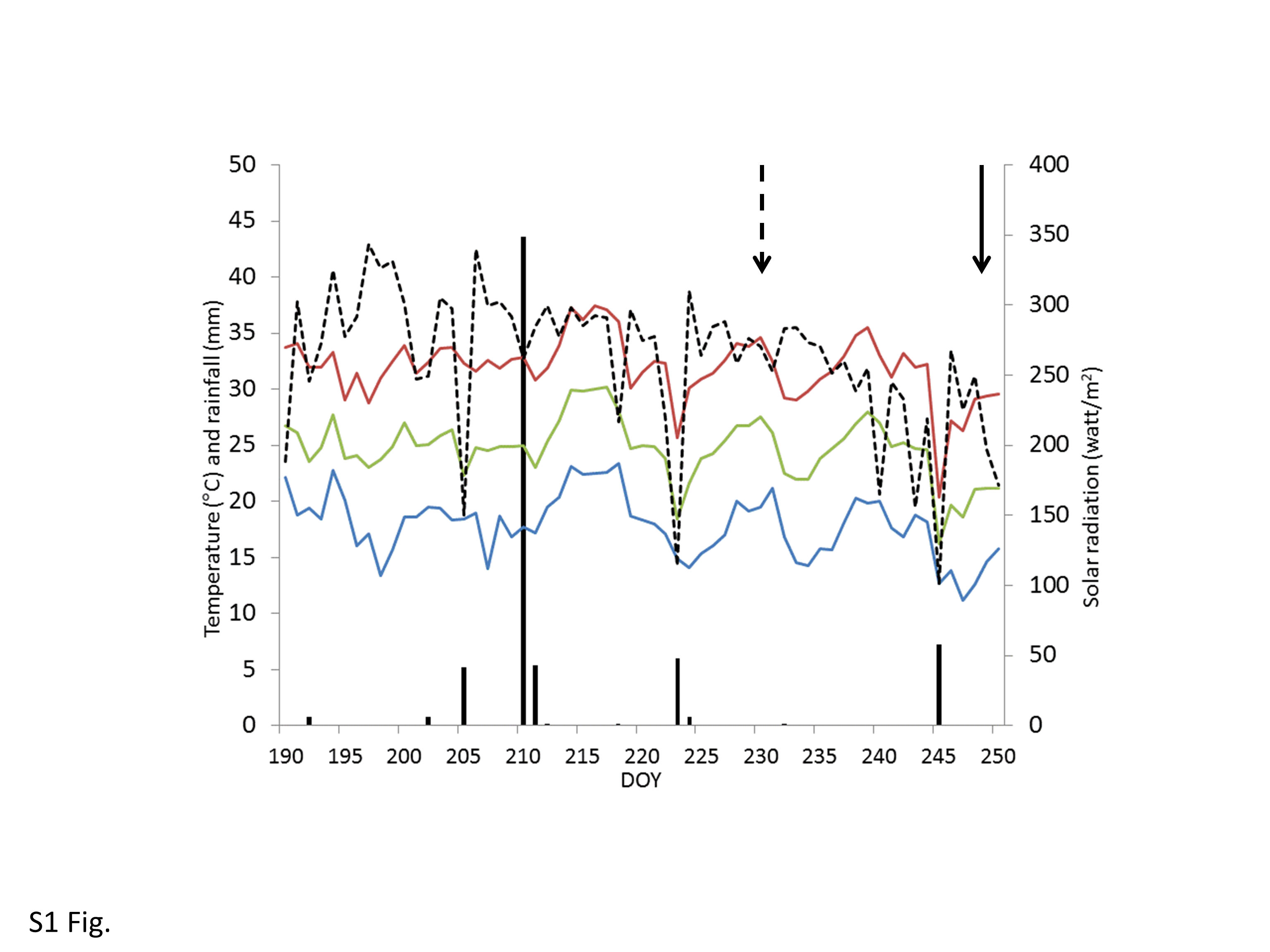

Supplement: S1 Fig — Arrows indicate harvest dates for Malvasia C.a., Malvasia R., Ortrugo (broken) and Barbera and Ervi (solid). (TIF) [file pone.0216421.s001.tif]

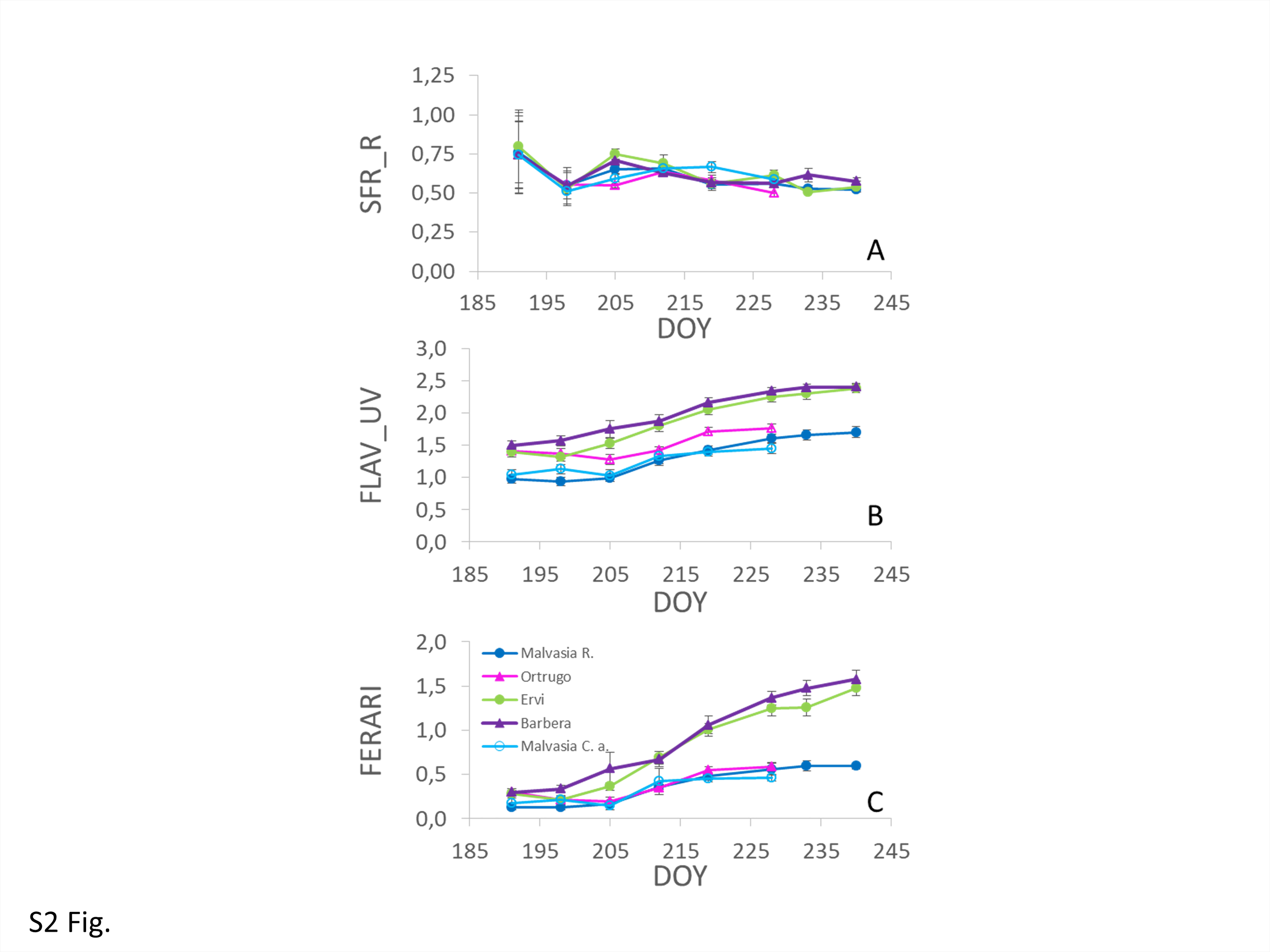

Supplement: S2 Fig — Within each date, Mx readings were pooled over cluster side as well as cluster and vine replicates (n = 24). (TIF) [file pone.0216421.s002.tif]

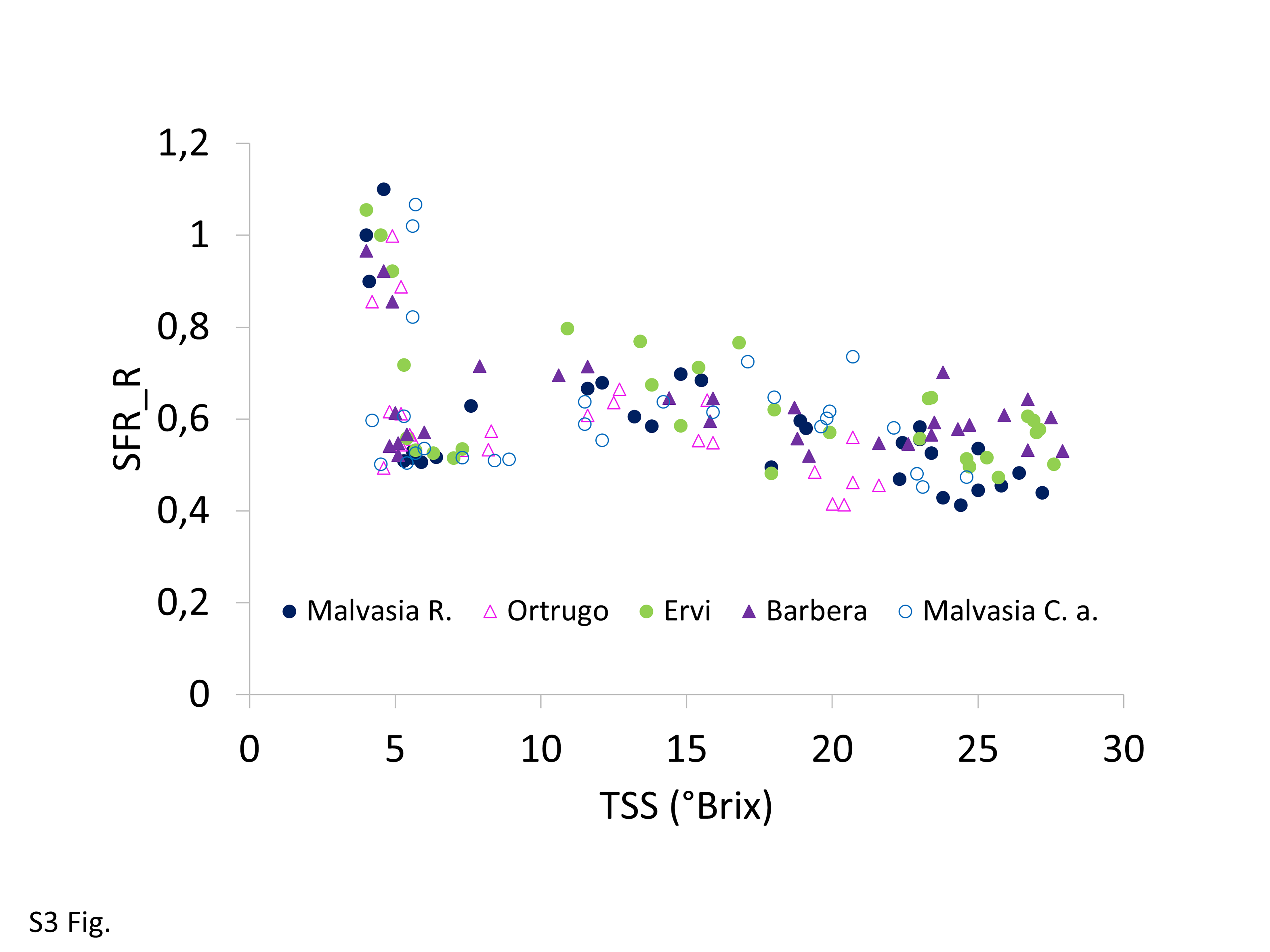

Supplement: S3 Fig — Equation of the linear model is: y = 1.0015x -0.6066. Data were pooled over cultivars and sampling dates. (TIF) [file pone.0216421.s003.tif]

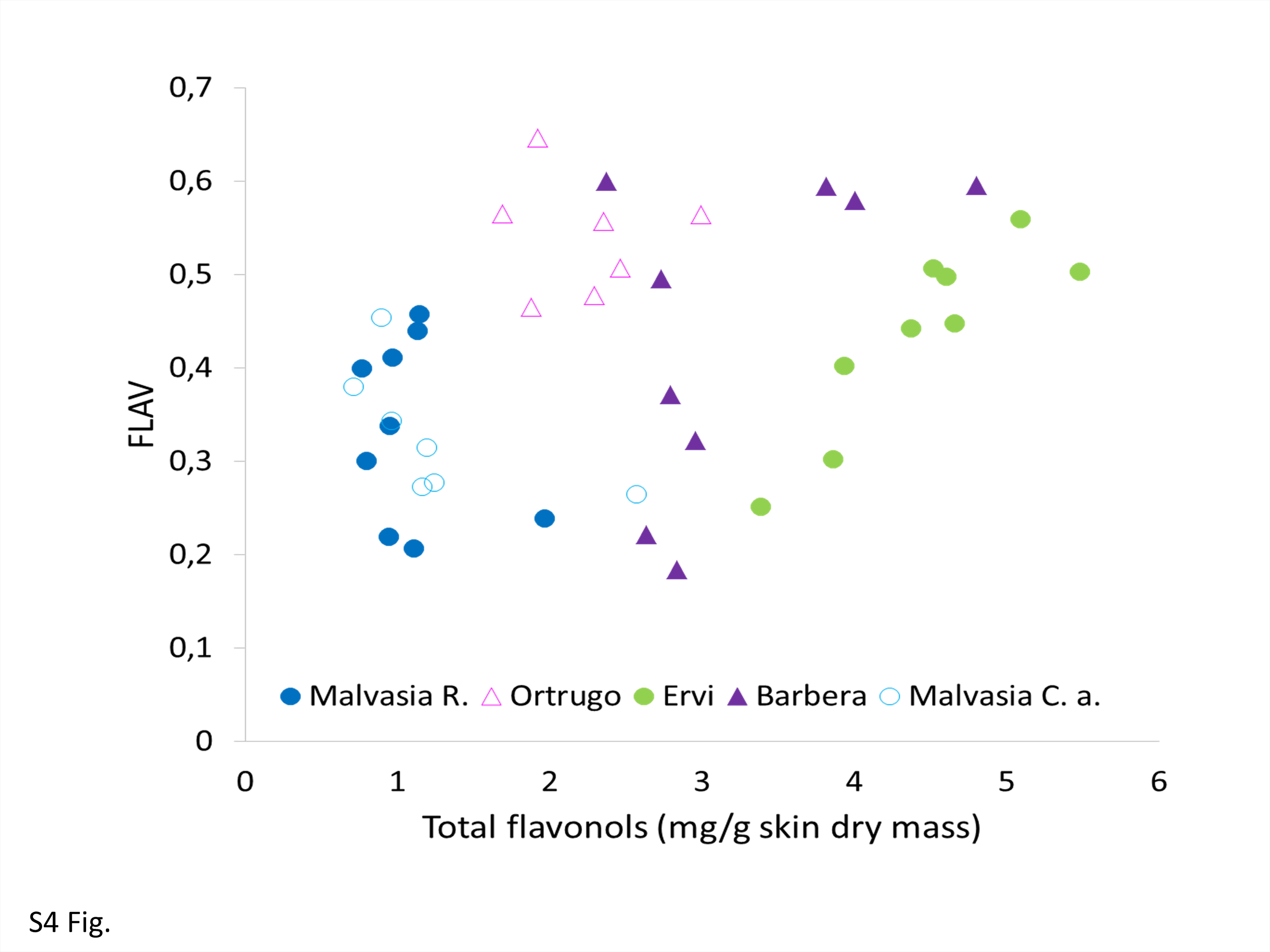

Supplement: S4 Fig — Data were pooled over sampling dates and cultivars. A clear inflexion point is visible across the 10 °Brix threshold. (TIF) [file pone.0216421.s004.tif]

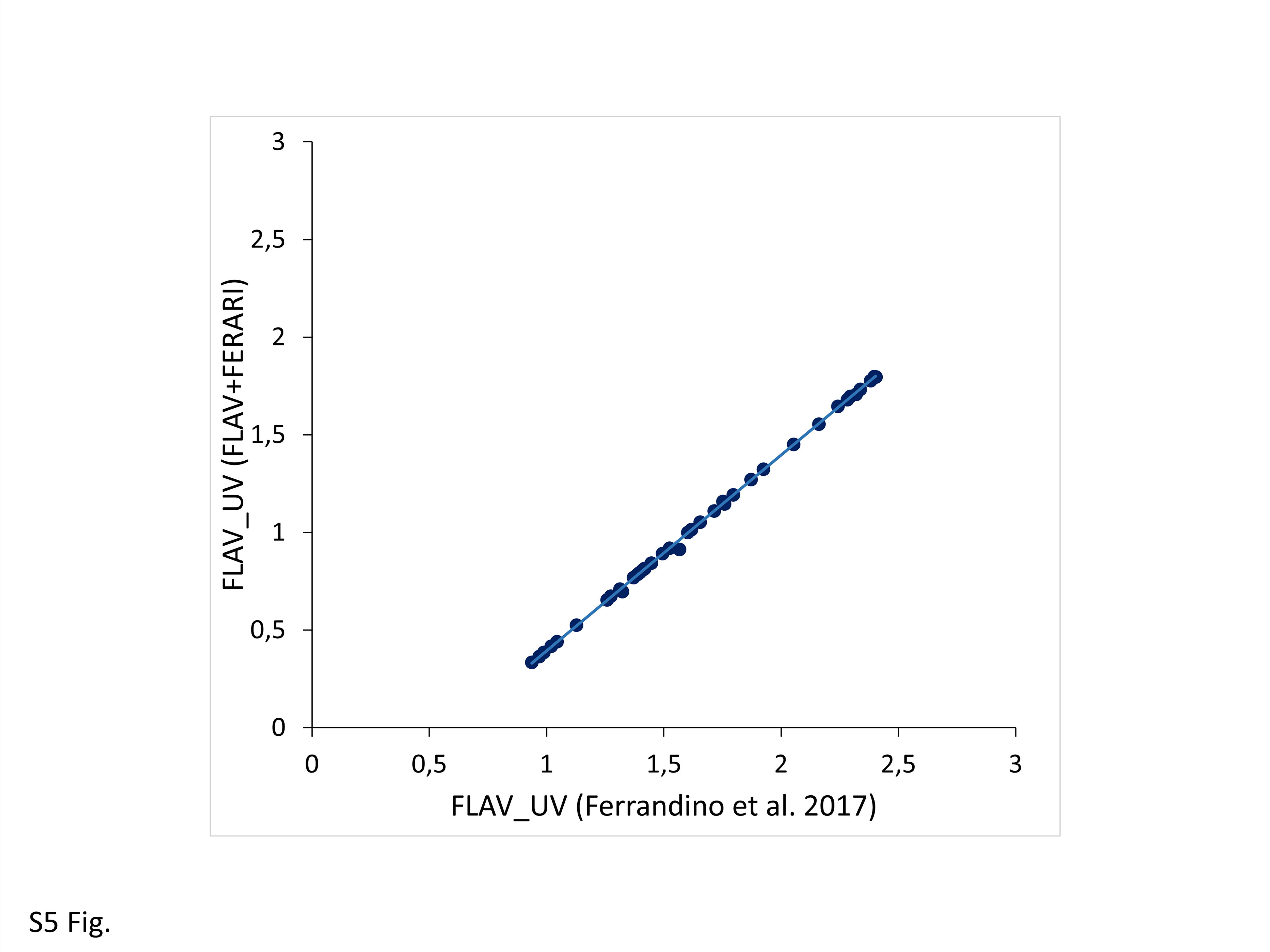

Supplement: S5 Fig — Data represent all sampling dates x cultivar combinations. (TIF) [file pone.0216421.s005.tif]
