# Supplementary material for: Destructive and optical non-destructive grape ripening assessment: Agronomic comparison and cost-benefit analysis
Source: PLoS One. 2019 May 29;14(5):e0216421. doi: 10.1371/journal.pone.0216421 (PMC6541254; doi:10.1371/journal.pone.0216421)
Supplement: S1 Table — (DOCX) [file pone.0216421.s006.docx]

|  |  | Skin to berry ratio (%) | | | | | | | |
| --- | --- | --- | --- | --- | --- | --- | --- | --- | --- |
|  | ***DOY*** | 190 | 199 | 205 | 213 | 219 | 228 | 233 | 240 |
| ***Cultivar*** |  |  |  |  |  |  |  |  |  |
| Ortrugo |  | 9.86 | 10.67 | 10.52 | 10.93 | 10.46 | 9.70 | - | - |
| Malvasia C. a. |  | 10.87 | 10.30 | 10.57 | 9.55 | 10.50 | 10.31 | - | - |
| Malvasia R. |  | 10.70 | 9.93 | 10.37 | 9.95 | 8.61 | 10.30 | 11.11 | 13.79 |
| Barbera |  | 7.58 | 7.28 | 8.35 | 10.67 | 11.86 | 8.84 | 10.37 | 10.25 |
| Ervi |  | 8.18 | 7.73 | 8.19 | 8.73 | 8.55 | 8.47 | 7.79 | 9.08 |
