# Supplementary material for: Destructive and optical non-destructive grape ripening assessment: Agronomic comparison and cost-benefit analysis
Source: PLoS One. 2019 May 29;14(5):e0216421. doi: 10.1371/journal.pone.0216421 (PMC6541254; doi:10.1371/journal.pone.0216421)
Supplement: S1 Data — (DOCX) [file pone.0216421.s007.docx]

|  |  |  |  |  |  |  |
| --- | --- | --- | --- | --- | --- | --- |
|  |  |  |  |  |  |  |
| Fig.1 - Fresh Berry mass |  | Means | S.E. | Statistical Elaboration | p Value | # samples |
| CULTIVAR | DOY |  |  | Means with standard errors | - | 3 |
| Malvasia R. |  |  |  |  |  |  |
|  | 191 | 0,8902 | 0,039807 |  |  |  |
|  | 199 | 1,064267 | 0,11114 |  |  |  |
|  | 205 | 1,285267 | 0,056032 |  |  |  |
|  | 213 | 1,784533 | 0,086037 |  |  |  |
|  | 219 | 1,899133 | 0,174692 |  |  |  |
|  | 228 | 1,907267 | 0,084552 |  |  |  |
|  | 233 | 1,938267 | 0,058641 |  |  |  |
|  | 240 | 1,973733 | 0,160659 |  |  |  |
| Ortrugo |  |  |  |  |  |  |
|  | 191 | 0,8822 | 0,060046 |  |  |  |
|  | 199 | 0,838733 | 0,056743 |  |  |  |
|  | 205 | 0,780467 | 0,026712 |  |  |  |
|  | 213 | 1,045467 | 0,041308 |  |  |  |
|  | 219 | 1,212933 | 0,082242 |  |  |  |
|  | 228 | 1,556 | 0,160316 |  |  |  |
| Ervi |  |  |  |  |  |  |
|  | 191 | 0,904733 | 0,056844 |  |  |  |
|  | 199 | 0,960667 | 0,036668 |  |  |  |
|  | 205 | 1,070467 | 0,045665 |  |  |  |
|  | 213 | 1,3078 | 0,039055 |  |  |  |
|  | 219 | 1,415267 | 0,014155 |  |  |  |
|  | 228 | 1,487067 | 0,02622 |  |  |  |
|  | 233 | 1,568667 | 0,032966 |  |  |  |
|  | 240 | 1,473067 | 0,038637 |  |  |  |
| Barbera |  |  |  |  |  |  |
|  | 191 | 0,946467 | 0,033998 |  |  |  |
|  | 199 | 1,087667 | 0,008042 |  |  |  |
|  | 205 | 1,026 | 0,104929 |  |  |  |
|  | 213 | 1,436 | 0,060497 |  |  |  |
|  | 219 | 1,595333 | 0,040251 |  |  |  |
|  | 228 | 1,7098 | 0,026598 |  |  |  |
|  | 233 | 1,695933 | 0,113002 |  |  |  |
|  | 240 | 1,5956 | 0,050475 |  |  |  |
| Malvasia C. a. |  |  |  |  |  |  |
|  | 191 | 1 | 0 |  |  |  |
|  | 199 | 1 | 0,105795 |  |  |  |
|  | 205 | 1 | 0,076872 |  |  |  |
|  | 213 | 1,565333 | 0,093871 |  |  |  |
|  | 219 | 1,708667 | 0,046646 |  |  |  |
|  | 228 | 1,738 | 0,063318 |  |  |  |
|  |  |  |  |  |  |  |
|  |  |  |  |  |  |  |
| Fig.2a TSS |  | Means | S.E. | Statistical Elaboration | p Value | # samples |
| CULTIVAR | DOY |  |  | Means with standard errors | - | 3 |
| Malvasia R. |  |  |  |  |  |  |
|  | 191 | 5,5 | 0,1 |  |  |  |
|  | 199 | 6,633333 | 0,504425 |  |  |  |
|  | 205 | 12,3 | 0,472582 |  |  |  |
|  | 213 | 14,7 | 0,493288 |  |  |  |
|  | 219 | 18,63333 | 0,371184 |  |  |  |
|  | 228 | 22,8 | 0,2 |  |  |  |
|  | 233 | 23,56667 | 0,783865 |  |  |  |
|  | 240 | 25,06667 | 0,751295 |  |  |  |
| Ortrugo |  |  |  |  |  |  |
|  | 191 | 4,866667 | 0,176383 |  |  |  |
|  | 199 | 5,433333 | 0,120185 |  |  |  |
|  | 205 | 7,933333 | 0,31798 |  |  |  |
|  | 213 | 12,26667 | 0,338296 |  |  |  |
|  | 219 | 15,66667 | 0,145297 |  |  |  |
|  | 228 | 20,26667 | 0,433333 |  |  |  |
| Ervi |  |  |  |  |  |  |
|  | 191 | 5,466667 | 0,120185 |  |  |  |
|  | 199 | 6,866667 | 0,296273 |  |  |  |
|  | 205 | 12,7 | 0,907377 |  |  |  |
|  | 213 | 15,66667 | 0,592546 |  |  |  |
|  | 219 | 18,6 | 0,650641 |  |  |  |
|  | 228 | 23,23333 | 0,120185 |  |  |  |
|  | 233 | 24,86667 | 0,218581 |  |  |  |
|  | 240 | 26,6 | 0,450925 |  |  |  |
| Barbera |  |  |  |  |  |  |
|  | 191 | 5,066667 | 0,176383 |  |  |  |
|  | 199 | 5,4 | 0,3 |  |  |  |
|  | 205 | 10,03333 | 1,105039 |  |  |  |
|  | 213 | 15,36667 | 0,484195 |  |  |  |
|  | 219 | 18,9 | 0,152753 |  |  |  |
|  | 228 | 22,56667 | 0,548736 |  |  |  |
|  | 233 | 23,83333 | 0,260342 |  |  |  |
|  | 240 | 25,76667 | 0,581187 |  |  |  |
| Malvasia C. a. |  |  |  |  |  |  |
|  | 191 | 5,633333 | 0,033333 |  |  |  |
|  | 199 | 5,7 | 0,173205 |  |  |  |
|  | 205 | 8,2 | 0,472582 |  |  |  |
|  | 213 | 11,7 | 0,2 |  |  |  |
|  | 219 | 15,73333 | 0,841295 |  |  |  |
|  | 228 | 19,53333 | 0,800694 |  |  |  |
|  |  |  |  |  |  |  |
| Fig.2b pH |  | Means | S.E. | Statistical Elaboration | p Value | # samples |
| CULTIVAR | DOY |  |  | Means with standard errors | - | 3 |
| Malvasia R. |  |  |  |  |  |  |
|  | 191 | 2,48 | 0,015275 |  |  |  |
|  | 199 | 2,363333 | 0,018559 |  |  |  |
|  | 205 | 2,64 | 0,011547 |  |  |  |
|  | 213 | 2,853333 | 0,023333 |  |  |  |
|  | 219 | 3,08 | 0,015275 |  |  |  |
|  | 228 | 3,21 | 0,032146 |  |  |  |
|  | 233 | 3,28 | 0,011547 |  |  |  |
|  | 240 | 3,366667 | 0,027285 |  |  |  |
| Ortrugo |  |  |  |  |  |  |
|  | 191 | 2,493333 | 0,003333 |  |  |  |
|  | 199 | 2,37 | 0,023094 |  |  |  |
|  | 205 | 2,486667 | 0,020276 |  |  |  |
|  | 213 | 2,653333 | 0,008819 |  |  |  |
|  | 219 | 2,836667 | 0,017638 |  |  |  |
|  | 228 | 3,05 | 0,015275 |  |  |  |
| Ervi |  |  |  |  |  |  |
|  | 191 | 2,456667 | 0,018559 |  |  |  |
|  | 199 | 2,27 | 0,026458 |  |  |  |
|  | 205 | 2,503333 | 0,017638 |  |  |  |
|  | 213 | 2,776667 | 0,008819 |  |  |  |
|  | 219 | 2,88 | 0,020817 |  |  |  |
|  | 228 | 3,096667 | 0,006667 |  |  |  |
|  | 233 | 3,15 | 0,011547 |  |  |  |
|  | 240 | 3,216667 | 0,020276 |  |  |  |
| Barbera |  |  |  |  |  |  |
|  | 191 | 2,46 | 0,035119 |  |  |  |
|  | 199 | 2,28 | 0,03 |  |  |  |
|  | 205 | 2,486667 | 0,043716 |  |  |  |
|  | 213 | 2,77 | 0,040415 |  |  |  |
|  | 219 | 2,8 | 0,023094 |  |  |  |
|  | 228 | 2,966667 | 0,021858 |  |  |  |
|  | 233 | 3 | 0,02848 |  |  |  |
|  | 240 | 3,046667 | 0,027285 |  |  |  |
| Malvasia C. a. |  |  |  |  |  |  |
|  | 191 | 2,576667 | 0,008819 |  |  |  |
|  | 199 | 2,343333 | 0,01453 |  |  |  |
|  | 205 | 2,45 | 0,032146 |  |  |  |
|  | 213 | 2,743333 | 0,013333 |  |  |  |
|  | 219 | 2,883333 | 0,049103 |  |  |  |
|  | 228 | 3,05 | 0,037859 |  |  |  |
|  |  |  |  |  |  |  |
| Fig.2c Titratable acidity |  | Means | S.E. | Statistical Elaboration | p Value | # samples |
| CULTIVAR | DOY |  |  | Means with standard errors | - | 3 |
| Malvasia R. |  |  |  |  |  |  |
|  | 191 | 47,09 | 0,963656 |  |  |  |
|  | 199 | 43,86667 | 1,996466 |  |  |  |
|  | 205 | 20,51667 | 1,338859 |  |  |  |
|  | 213 | 13,58 | 0,879564 |  |  |  |
|  | 219 | 7,983333 | 0,06888 |  |  |  |
|  | 228 | 6,46 | 0,228983 |  |  |  |
|  | 233 | 5,53 | 0,108167 |  |  |  |
|  | 240 | 4,83 | 0,334714 |  |  |  |
| Ortrugo |  |  |  |  |  |  |
|  | 191 | 39,71667 | 0,39918 |  |  |  |
|  | 199 | 35,49 | 0,408534 |  |  |  |
|  | 205 | 28,23333 | 0,989281 |  |  |  |
|  | 213 | 18,9 | 0,295127 |  |  |  |
|  | 219 | 9,186667 | 0,503466 |  |  |  |
|  | 228 | 6,113333 | 0,47876 |  |  |  |
| Ervi |  |  |  |  |  |  |
|  | 191 | 48,12667 | 0,424356 |  |  |  |
|  | 199 | 46,7 | 0,42194 |  |  |  |
|  | 205 | 29,62 | 1,902919 |  |  |  |
|  | 213 | 18,62667 | 0,369745 |  |  |  |
|  | 219 | 13,62333 | 0,543732 |  |  |  |
|  | 228 | 9,74 | 0,091652 |  |  |  |
|  | 233 | 9,006667 | 0,341581 |  |  |  |
|  | 240 | 7,75 | 0,05 |  |  |  |
| Barbera |  |  |  |  |  |  |
|  | 191 | 46,33667 | 1,369676 |  |  |  |
|  | 199 | 44,12 | 0,111355 |  |  |  |
|  | 205 | 35,6 | 2,057482 |  |  |  |
|  | 213 | 19,41667 | 1,629032 |  |  |  |
|  | 219 | 15,32667 | 0,594652 |  |  |  |
|  | 228 | 11,20333 | 0,417306 |  |  |  |
|  | 233 | 10,62333 | 0,381765 |  |  |  |
|  | 240 | 9,886667 | 0,080069 |  |  |  |
| Malvasia C. a. |  |  |  |  |  |  |
|  | 191 | 41,28 | 0,92376 |  |  |  |
|  | 199 | 41,73333 | 1,099338 |  |  |  |
|  | 205 | 34,07667 | 2,142229 |  |  |  |
|  | 213 | 16,63 | 0,415812 |  |  |  |
|  | 219 | 9,243333 | 0,576927 |  |  |  |
|  | 228 | 6,893333 | 0,311787 |  |  |  |
|  |  |  |  |  |  |  |
| Fig.2d Malic Acid |  | Means | S.E. | Statistical Elaboration | p Value | # samples |
| CULTIVAR | DOY |  |  | Means with standard errors | - | 3 |
| Malvasia R. |  |  |  |  |  |  |
|  | 191 | 20,53035 | 6,556145 |  |  |  |
|  | 199 | 25,82992 | 0,499515 |  |  |  |
|  | 205 | 10,94091 | 0,545854 |  |  |  |
|  | 213 | 5,701508 | 0,379166 |  |  |  |
|  | 219 | 2,634181 | 0,065568 |  |  |  |
|  | 228 | 1,923853 | 0,088642 |  |  |  |
|  | 233 | 1,181236 | 0,220529 |  |  |  |
|  | 240 | 0,445959 | 0,021916 |  |  |  |
| Ortrugo |  |  |  |  |  |  |
|  | 191 | 19,80681 | 1,343696 |  |  |  |
|  | 199 | 17,40578 | 0,323347 |  |  |  |
|  | 205 | 7,333502 | 2,070644 |  |  |  |
|  | 213 | 5,628127 | 0,31823 |  |  |  |
|  | 219 | 1,4322 | 0,349811 |  |  |  |
|  | 228 | 0,564836 | 0,180004 |  |  |  |
| Ervi |  |  |  |  |  |  |
|  | 191 | 26,07795 | 0,513502 |  |  |  |
|  | 199 | 25,47916 | 0,52737 |  |  |  |
|  | 205 | 14,43238 | 0,153806 |  |  |  |
|  | 213 | 9,010992 | 0,252213 |  |  |  |
|  | 219 | 4,94128 | 0,246092 |  |  |  |
|  | 228 | 3,099417 | 0,356342 |  |  |  |
|  | 233 | 1,988428 | 0,179932 |  |  |  |
|  | 240 | 1,521724 | 0,274727 |  |  |  |
| Barbera |  |  |  |  |  |  |
|  | 191 | 23,01796 | 0,669626 |  |  |  |
|  | 199 | 23,31589 | 0,042333 |  |  |  |
|  | 205 | 15,07667 | 1,777532 |  |  |  |
|  | 213 | 8,566303 | 1,418638 |  |  |  |
|  | 219 | 4,763698 | 0,46985 |  |  |  |
|  | 228 | 2,346527 | 0,803604 |  |  |  |
|  | 233 | 2,136657 | 0,406888 |  |  |  |
|  | 240 | 1,668487 |  |  |  |  |
| Malvasia C. a. |  |  |  |  |  |  |
|  | 191 | 20,9134 | 0,780591 |  |  |  |
|  | 199 | 21,50485 | 0,664732 |  |  |  |
|  | 205 | 16,43422 | 1,496564 |  |  |  |
|  | 213 | 6,900554 | 0,712853 |  |  |  |
|  | 219 | 2,268743 | 0,241694 |  |  |  |
|  | 228 | 1,308919 | 0,09379 |  |  |  |
|  |  |  |  |  |  |  |
| Fig. 3a Total anthocyanins |  | Means | S.E. | Statistical Elaboration | p Value | # samples |
| CULTIVAR | DOY |  |  | Means with standard errors | - | 3 |
| Malvasia R. |  |  |  |  |  |  |
|  | 191 | 0,047539 | 0,005556 |  |  |  |
|  | 199 | 0,029722 | 0,004096 |  |  |  |
|  | 205 | 0,033137 | 0,00374 |  |  |  |
|  | 213 | 0,137117 | 0,022898 |  |  |  |
|  | 219 | 0,206933 | 0,030715 |  |  |  |
|  | 228 | 0,225218 | 0,025673 |  |  |  |
|  | 233 | 0,181366 | 0,028829 |  |  |  |
|  | 240 | 0,22951 | 0,038832 |  |  |  |
| Ortrugo |  |  |  |  |  |  |
|  | 191 | 0,034881 | 0,001029 |  |  |  |
|  | 199 | 0,030668 | 0,00255 |  |  |  |
|  | 205 | 0,011737 | 0,003314 |  |  |  |
|  | 213 | 0,014603 | 0,003459 |  |  |  |
|  | 219 | 0,038462 | 0,001758 |  |  |  |
|  | 228 | 0,030345 | 0,00439 |  |  |  |
| Ervi |  |  |  |  |  |  |
|  | 191 | 0,006824 | 0,001405 |  |  |  |
|  | 199 | 0,017167 | 0,00397 |  |  |  |
|  | 205 | 0,269432 | 0,087059 |  |  |  |
|  | 213 | 0,60611 | 0,115621 |  |  |  |
|  | 219 | 1,003336 | 0,197715 |  |  |  |
|  | 228 | 1,664353 | 0,071121 |  |  |  |
|  | 233 | 1,768857 | 0,081043 |  |  |  |
|  | 240 | 2,005282 | 0,1099 |  |  |  |
| Barbera |  |  |  |  |  |  |
|  | 191 | 0,004617 | 0,001356 |  |  |  |
|  | 199 | 0,011222 | 0,002034 |  |  |  |
|  | 205 | 0,116719 | 0,029573 |  |  |  |
|  | 213 | 0,58563 | 0,079104 |  |  |  |
|  | 219 | 1,00925 | 0,032845 |  |  |  |
|  | 228 | 1,458173 | 0,052844 |  |  |  |
|  | 233 | 1,430536 | 0,059122 |  |  |  |
|  | 240 | 1,55999 | 0,10217 |  |  |  |
| Malvasia C. a. |  |  |  |  |  |  |
|  | 191 | 0,041099 | 0,004206 |  |  |  |
|  | 199 | 0,048774 | 0,00294 |  |  |  |
|  | 205 | 0,014256 | 0,001604 |  |  |  |
|  | 213 | 0,025803 | 0,001612 |  |  |  |
|  | 219 | 0,059676 | 0,003534 |  |  |  |
|  | 228 | 0,059239 | 0,001588 |  |  |  |
|  |  |  |  |  |  |  |
| Fig. 3b Total phenolics |  | Means | S.E. | Statistical Elaboration | p Value | # samples |
| CULTIVAR | DOY |  |  | Means with standard errors | - | 3 |
| Malvasia R. |  |  |  |  |  |  |
|  | 191 | 5,140349 | 0,618476 |  |  |  |
|  | 199 | 4,537792 | 0,217706 |  |  |  |
|  | 205 | 3,608405 | 0,265589 |  |  |  |
|  | 213 | 2,5644 | 0,038368 |  |  |  |
|  | 219 | 2,424639 | 0,128817 |  |  |  |
|  | 228 | 2,250788 | 0,136302 |  |  |  |
|  | 233 | 1,889473 | 0,140474 |  |  |  |
|  | 240 | 2,070184 | 0,230103 |  |  |  |
| Ortrugo |  |  |  |  |  |  |
|  | 191 | 4,930681 | 0,177944 |  |  |  |
|  | 199 | 5,05152 | 0,353438 |  |  |  |
|  | 205 | 4,169252 | 0,215546 |  |  |  |
|  | 213 | 3,689169 | 0,221432 |  |  |  |
|  | 219 | 2,514727 | 0,075733 |  |  |  |
|  | 228 | 2,071497 | 0,039411 |  |  |  |
| Ervi |  |  |  |  |  |  |
|  | 191 | 4,801958 | 0,150598 |  |  |  |
|  | 199 | 4,199286 | 0,045222 |  |  |  |
|  | 205 | 3,907027 | 0,261327 |  |  |  |
|  | 213 | 3,504636 | 0,194737 |  |  |  |
|  | 219 | 2,88762 | 0,208444 |  |  |  |
|  | 228 | 3,450334 | 0,132606 |  |  |  |
|  | 233 | 3,567628 | 0,179157 |  |  |  |
|  | 240 | 3,60892 | 0,190101 |  |  |  |
| Barbera |  |  |  |  |  |  |
|  | 191 | 3,681222 | 0,169425 |  |  |  |
|  | 199 | 5,006248 | 0,080914 |  |  |  |
|  | 205 | 3,365438 | 0,245792 |  |  |  |
|  | 213 | 2,727119 | 0,205317 |  |  |  |
|  | 219 | 2,316828 | 0,140367 |  |  |  |
|  | 228 | 2,641385 | 0,078814 |  |  |  |
|  | 233 | 2,584636 | 0,172511 |  |  |  |
|  | 240 | 2,693714 | 0,152953 |  |  |  |
| Malvasia C. a. |  |  |  |  |  |  |
|  | 191 | 5,9107 | 0,488369 |  |  |  |
|  | 199 | 5,442425 | 0,289243 |  |  |  |
|  | 205 | 4,177163 | 0,022158 |  |  |  |
|  | 213 | 3,425065 | 0,136024 |  |  |  |
|  | 219 | 2,504651 | 0,134383 |  |  |  |
|  | 228 | 2,300985 | 0,167679 |  |  |  |
|  |  |  |  |  |  |  |
| Fig. 3c Total flavonols |  | Means | S.E. | Statistical Elaboration | p Value | # samples |
| CULTIVAR | DOY |  |  | Means with standard errors | - | 3 |
| Malvasia R. |  |  |  |  |  |  |
|  | 191 | 1,964301 | 0,160688 |  |  |  |
|  | 199 | 1,100551 | 0,107217 |  |  |  |
|  | 205 | 0,940856 | 0,203935 |  |  |  |
|  | 213 | 0,793257 | 0,145508 |  |  |  |
|  | 219 | 0,942175 | 0,208032 |  |  |  |
|  | 228 | 1,129778 | 0,277922 |  |  |  |
|  | 233 | 1,141153 | 0,149721 |  |  |  |
|  | 240 | 0,961947 | 0,328516 |  |  |  |
| Ortrugo |  |  |  |  |  |  |
|  | 191 | 2,458853 | 0,217401 |  |  |  |
|  | 199 | 2,352083 | 0,267415 |  |  |  |
|  | 205 | 2,293065 | 0,193546 |  |  |  |
|  | 213 | 1,875702 | 0,416759 |  |  |  |
|  | 219 | 2,990946 | 0,851037 |  |  |  |
|  | 228 | 1,686086 | 0,246464 |  |  |  |
| Ervi |  |  |  |  |  |  |
|  | 191 | 5,475515 | 0,432234 |  |  |  |
|  | 199 | 4,59694 | 0,209779 |  |  |  |
|  | 205 | 5,085549 | 0,468212 |  |  |  |
|  | 213 | 4,516223 | 0,446581 |  |  |  |
|  | 219 | 4,652935 | 0,575681 |  |  |  |
|  | 228 | 3,929795 | 0,232592 |  |  |  |
|  | 233 | 4,366456 | 0,446295 |  |  |  |
|  | 240 | 3,854648 | 0,214741 |  |  |  |
| Barbera |  |  |  |  |  |  |
|  | 191 | 4,80363 | 0,351521 |  |  |  |
|  | 199 | 4,000705 | 0,311015 |  |  |  |
|  | 205 | 3,815867 | 0,422577 |  |  |  |
|  | 213 | 2,369583 | 0,044709 |  |  |  |
|  | 219 | 2,730189 | 0,282242 |  |  |  |
|  | 228 | 2,791857 | 0,191348 |  |  |  |
|  | 233 | 2,953307 | 0,310082 |  |  |  |
|  | 240 | 2,630183 | 0,413452 |  |  |  |
| Malvasia C. a. |  |  |  |  |  |  |
|  | 191 | 2,562744 | 0,083911 |  |  |  |
|  | 199 | 1,188587 | 0,090288 |  |  |  |
|  | 205 | 1,157553 | 0,026499 |  |  |  |
|  | 213 | 1,235835 | 0,143437 |  |  |  |
|  | 219 | 0,956503 | 0,208032 |  |  |  |
|  | 228 | 0,707805 | 0,065055 |  |  |  |

| Fig.4a - Anth_RG | X axis | Yaxis | Statistical Elaboration | # samples |
| --- | --- | --- | --- | --- |
|  | ANTH_RG SOUTH-WEST | ANTH_RG NORTH-EAST | Linear regression | 27 |
|  | -0,039766667 | -0,049733333 |  |  |
|  | -0,034091667 | -0,040366667 |  |  |
|  | 0,037433333 | -0,001225 |  |  |
|  | 0,263716667 | 0,216275 |  |  |
|  | 0,397958333 | 0,27895 |  |  |
|  | 0,334275 | 0,348525 |  |  |
|  | 0,385608333 | 0,353541667 |  |  |
|  | -0,026008333 | -0,0413 |  |  |
|  | -0,005883333 | -0,016891667 |  |  |
|  | 0,1055 | 0,072366667 |  |  |
|  | 0,100633333 | 0,108183333 |  |  |
|  | 0,0035 | 0,047016667 |  |  |
|  | -0,012 | 0,014658333 |  |  |
|  | 0,00475 | 0,034725 |  |  |
|  | -0,007541667 | 0,004991667 |  |  |
|  | -0,027083333 | -0,026666667 |  |  |
|  | -0,008833333 | -0,008233333 |  |  |
|  | 0,011183333 | 0,0099 |  |  |
|  | 0,051375 | 0,0296 |  |  |
|  | 0,0707 | 0,087891667 |  |  |
|  | 0,0491 | -0,002075 |  |  |
|  | 0,018375 | 0,01205 |  |  |
|  | 0,047291667 | 0,037325 |  |  |
|  | -0,014858333 | -0,004325 |  |  |
|  | 0,00475 | 0,034725 |  |  |
|  | 0,0035 | 0,047016667 |  |  |
|  | -0,039308333 | -0,007216667 |  |  |
|  |  |  |  |  |
| Fig.4b - FERARI | X axis | Yaxis | Statistical Elaboration | # samples |
|  | FERARI SOUTH-WEST | FERARI NORTH-EAST | Linear regression | 27 |
|  | 0,318841667 | 0,274008333 |  |  |
|  | 0,397716667 | 0,268366667 |  |  |
|  | 0,468291667 | 0,66055 |  |  |
|  | 0,719716667 | 0,622658333 |  |  |
|  | 1,039466667 | 1,077841667 |  |  |
|  | 1,380825 | 1,342791667 |  |  |
|  | 1,540741667 | 1,41175 |  |  |
|  | 1,693166667 | 1,458258333 |  |  |
|  | 1,58125 | 1,465833333 |  |  |
|  | 0,279016667 | 0,284575 |  |  |
|  | 0,262933333 | 0,159125 |  |  |
|  | 0,403275 | 0,318441667 |  |  |
|  | 0,710841667 | 0,660666667 |  |  |
|  | 1,0106 | 0,996191667 |  |  |
|  | 1,211316667 | 1,272866667 |  |  |
|  | 1,235691667 | 1,27075 |  |  |
|  | 1,513558333 | 1,43625 |  |  |
|  | 1,586833333 | 1,269466667 |  |  |
|  | 0,09065 | 0,161425 |  |  |
|  | 0,138483333 | 0,115208333 |  |  |
|  | 0,196825 | 0,13425 |  |  |
|  | 0,343566667 | 0,363866667 |  |  |
|  | 0,473508333 | 0,479408333 |  |  |
|  | 0,538341667 | 0,579433333 |  |  |
|  | 1,513558333 | 1,43625 |  |  |
|  | 0,397716667 | 0,268366667 |  |  |
|  | 0,60145 | 0,587691667 |  |  |
|  |  |  |  |  |
|  |  |  |  |  |
| Fig.4c- FLAV_UV | X axis | Yaxis | Statistical Elaboration | # samples |
|  | FLAV_UV SOUTH-WEST | FLAV_UV NORTH-EAST | Linear regression | 41 |
|  | 1,55983198 | 1,428171393 |  |  |
|  | 1,682459351 | 1,449585165 |  |  |
|  | 1,727273892 | 1,773811819 |  |  |
|  | 1,885192288 | 1,857979801 |  |  |
|  | 2,178353669 | 2,140842222 |  |  |
|  | 2,338266859 | 2,333995696 |  |  |
|  | 2,392912116 | 2,399010109 |  |  |
|  | 2,426040656 | 2,379308884 |  |  |
|  | 2,339728653 | 2,296759188 |  |  |
|  | 1,457185101 | 1,317836109 |  |  |
|  | 1,372889334 | 1,24946645 |  |  |
|  | 1,601261209 | 1,444237239 |  |  |
|  | 1,829748741 | 1,760248533 |  |  |
|  | 2,07559978 | 2,030633668 |  |  |
|  | 2,242234071 | 2,240360025 |  |  |
|  | 2,273921342 | 2,313747408 |  |  |
|  | 2,367122008 | 2,392014607 |  |  |
|  | 2,339833128 | 2,22437934 |  |  |
|  | 0,984574046 | 1,103127746 |  |  |
|  | 1,129626626 | 1,125391839 |  |  |
|  | 0,999599971 | 1,040595969 |  |  |
|  | 1,434955117 | 1,210582118 |  |  |
|  | 1,422147823 | 1,372429242 |  |  |
|  | 1,490510818 | 1,401069153 |  |  |
|  | 1,693235919 | 1,540120371 |  |  |
|  | 0,879476986 | 1,055861766 |  |  |
|  | 0,913269941 | 0,95916322 |  |  |
|  | 0,970210859 | 1,004671416 |  |  |
|  | 1,212039324 | 1,30238555 |  |  |
|  | 1,371671238 | 1,463143454 |  |  |
|  | 1,545045944 | 1,657318015 |  |  |
|  | 1,603184289 | 1,704722114 |  |  |
|  | 1,693235919 | 1,540120371 |  |  |
|  | 1,129626626 | 1,125391839 |  |  |
|  | 1,461874202 | 1,360570039 |  |  |
|  | 1,449905708 | 1,290811539 |  |  |
|  | 1,286574092 | 1,260512441 |  |  |
|  | 1,421076691 | 1,411035632 |  |  |
|  | 1,769358719 | 1,656752224 |  |  |
|  | 1,855829704 | 1,659524181 |  |  |
|  | 1,968150478 | 1,880806318 |  |  |
|  |  |  |  |  |
| Fig.4D - SFR_R | X axis | Y axis | Statistical Elaboration | # samples |
|  | SFR_R SOUTH-WEST | SFR_R NORTH-EAST | Linear regression | 41 |
|  | 1,008816667 | 1,007091667 |  |  |
|  | 0,9087 | 0,848316667 |  |  |
|  | 0,79255 | 0,808208333 |  |  |
|  | 0,713266667 | 0,669666667 |  |  |
|  | 0,681641667 | 0,644616667 |  |  |
|  | 0,806441667 | 0,62485 |  |  |
|  | 0,698616667 | 0,60515 |  |  |
|  | 1,084383333 | 0,996141667 |  |  |
|  | 1,026816667 | 0,81055 |  |  |
|  | 0,8856 | 0,818075 |  |  |
|  | 0,770241667 | 0,720858333 |  |  |
|  | 0,71325 | 0,631941667 |  |  |
|  | 0,6333 | 0,60415 |  |  |
|  | 0,60095 | 0,548266667 |  |  |
|  | 1,067075 | 0,8372 |  |  |
|  | 1,0539 | 0,952858333 |  |  |
|  | 0,939958333 | 0,782658333 |  |  |
|  | 0,838691667 | 0,683691667 |  |  |
|  | 0,871958333 | 0,639191667 |  |  |
|  | 0,753391667 | 0,636375 |  |  |
|  | 0,792166667 | 0,59025 |  |  |
|  | 0,725158333 | 0,641408333 |  |  |
|  | 0,67855 | 0,610308333 |  |  |
|  | 1,10075 | 0,91125 |  |  |
|  | 1,047033333 | 0,92445 |  |  |
|  | 0,937575 | 0,755391667 |  |  |
|  | 0,872858333 | 0,8031 |  |  |
|  | 1,071133333 | 0,835333333 |  |  |
|  | 0,86415 | 0,760066667 |  |  |
|  | 0,782591667 | 0,718258333 |  |  |
|  | 0,704991667 | 0,823266667 |  |  |
|  | 0,896258333 | 0,726816667 |  |  |
|  | 1,090741667 | 0,970758333 |  |  |
|  | 1,205083333 | 0,978675 |  |  |
|  | 0,977283333 | 0,854 |  |  |
|  | 0,8747 | 0,744475 |  |  |
|  | 0,823441667 | 0,741466667 |  |  |
|  | 0,786741667 | 0,685466667 |  |  |
|  | 1,047033333 | 0,92445 |  |  |
|  | 0,871958333 | 0,639191667 |  |  |
|  | 0,712375 | 0,669641667 |  |  |
|  |  |  |  |  |
| Fig.5A - SFR_RxTSS | X axis | Y axis | Statistical Elaboration | # samples |
| CULTIVAR | TSS | SFR_R | Linear regression | 12 |
| Ortrugo | 12,7 | 0,6651 |  |  |
|  | 11,6 | 0,6083 |  |  |
|  | 12,5 | 0,6362 |  |  |
|  | 15,9 | 0,5489 |  |  |
|  | 15,4 | 0,553 |  |  |
|  | 15,7 | 0,6417 |  |  |
|  | 20,7 | 0,4623 |  |  |
|  | 19,4 | 0,4846 |  |  |
|  | 20,7 | 0,5601 |  |  |
|  | 20 | 0,4154 |  |  |
|  | 20,4 | 0,4139 |  |  |
|  | 21,6 | 0,4555 |  |  |
| Malvasia C.a. |  |  |  |  |
|  | 12,1 | 0,7252 |  |  |
|  | 11,5 | 0,6378 |  |  |
|  | 11,5 | 0,6153 |  |  |
|  | 20,7 | 0,602 |  |  |
|  | 18 | 0,5834 |  |  |
|  | 19,9 | 0,5813 |  |  |
|  | 19,8 | 0,4736 |  |  |
|  | 19,6 | 0,4814 |  |  |
|  | 22,1 | 0,4523 |  |  |
|  | 24,6 | 0,4783 |  |  |
|  | 22,9 | 0,5036 |  |  |
|  | 23,1 | 0,4514 |  |  |
|  |  |  |  |  |
|  |  | |  |  |
| Fig.5B - SFR_RxTSS | X axis | Y axis | Statistical Elaboration | # samples |
| CULTIVAR | TSS | SFR_R | Linear regression | 21 |
| Malvasia R | 13,2 | 0,6049 |  |  |
|  | 12,1 | 0,679 |  |  |
|  | 11,6 | 0,6661 |  |  |
|  | 15,5 | 0,6845 |  |  |
|  | 14,8 | 0,6981 |  |  |
|  | 13,8 | 0,5845 |  |  |
|  | 19,1 | 0,58 |  |  |
|  | 18,9 | 0,5963 |  |  |
|  | 17,9 | 0,4956 |  |  |
|  | 23 | 0,5829 |  |  |
|  | 23 | 0,5562 |  |  |
|  | 22,4 | 0,5484 |  |  |
|  | 25 | 0,5356 |  |  |
|  | 23,4 | 0,5265 |  |  |
|  | 22,3 | 0,4691 |  |  |
|  | 26,4 | 0,4825 |  |  |
|  | 25 | 0,4453 |  |  |
|  | 23,8 | 0,429 |  |  |
|  | 27,2 | 0,4398 |  |  |
|  | 25,8 | 0,4553 |  |  |
|  | 24,4 | 0,4126 |  |  |
| Ervi |  |  |  |  |
|  | 13,4 | 0,7694 |  |  |
|  | 13,8 | 0,6749 |  |  |
|  | 10,9 | 0,7974 |  |  |
|  | 16,8 | 0,7661 |  |  |
|  | 15,4 | 0,7122 |  |  |
|  | 14,8 | 0,5855 |  |  |
|  | 19,9 | 0,5712 |  |  |
|  | 18 | 0,6207 |  |  |
|  | 17,9 | 0,4818 |  |  |
|  | 23,4 | 0,6465 |  |  |
|  | 23,3 | 0,6451 |  |  |
|  | 23 | 0,5574 |  |  |
|  | 25,3 | 0,516 |  |  |
|  | 24,7 | 0,4963 |  |  |
|  | 24,6 | 0,5135 |  |  |
|  | 27,1 | 0,5771 |  |  |
|  | 27 | 0,571 |  |  |
|  | 25,7 | 0,4727 |  |  |
|  | 27,6 | 0,5014 |  |  |
|  | 26,9 | 0,597 |  |  |
|  | 26,7 | 0,6066 |  |  |
| Barbera |  |  |  |  |
|  | 11,6 | 0,7141 |  |  |
|  | 10,6 | 0,695 |  |  |
|  | 15,9 | 0,645 |  |  |
|  | 15,8 | 0,5954 |  |  |
|  | 15,9 | 0,645 |  |  |
|  | 14,4 | 0,6462 |  |  |
|  | 18,8 | 0,558 |  |  |
|  | 19,2 | 0,52 |  |  |
|  | 18,7 | 0,6247 |  |  |
|  | 21,6 | 0,548 |  |  |
|  | 23,5 | 0,5927 |  |  |
|  | 22,6 | 0,5459 |  |  |
|  | 23,8 | 0,7017 |  |  |
|  | 24,3 | 0,5779 |  |  |
|  | 23,4 | 0,566 |  |  |
|  | 24,7 | 0,5873 |  |  |
|  | 26,7 | 0,5321 |  |  |
|  | 25,9 | 0,6092 |  |  |
|  | 26,7 | 0,6434 |  |  |
|  | 27,9 | 0,5304 |  |  |
|  | 27,5 | 0,6036 |  |  |
|  |  |  |  |  |
|  |  |  |  |  |
| Fig.6 - FLAV_UV x Total Flavonols | X axis | Y axis | Statistical Elaboration | # samples |
| CULTIVAR | Total Flavonols | FLAV_UV | Polynomial model | 41 |
| Malvasia R. | 1,96430084 | 0,967669376 |  |  |
|  | 1,100550715 | 0,936216581 |  |  |
|  | 0,940856182 | 0,987441137 |  |  |
|  | 0,793257441 | 1,257212437 |  |  |
|  | 0,942175095 | 1,417407346 |  |  |
|  | 1,129778235 | 1,601181979 |  |  |
|  | 1,14115272 | 1,653953202 |  |  |
|  | 0,961946657 | 0,91 |  |  |
| Ortrugo |  |  |  |  |
|  | 0,760453134 | 1,1 |  |  |
|  | 2,458853074 | 1,41122212 |  |  |
|  | 2,352082718 | 1,370358624 |  |  |
|  | 2,293064875 | 1,273543266 |  |  |
|  | 1,87570158 | 1,416056162 |  |  |
|  | 2,990946347 | 1,713055471 |  |  |
|  | 1,686085944 | 1,757676943 |  |  |
|  | 1,917836965 | 1,924478398 |  |  |
| Ervi |  |  |  |  |
|  | 5,475515354 | 1,387510605 |  |  |
|  | 4,596939652 | 1,311177892 |  |  |
|  | 5,085549225 | 1,522749224 |  |  |
|  | 4,516222909 | 1,794998637 |  |  |
|  | 4,652935399 | 2,053116724 |  |  |
|  | 3,929795489 | 2,241297048 |  |  |
|  | 4,366456359 | 2,293834375 |  |  |
|  | 3,854648482 | 2,379568308 |  |  |
|  | 3,384045198 | 2,282106234 |  |  |
| Barbera |  |  |  |  |
|  | 4,80362963 | 1,494001687 |  |  |
|  | 4,000704922 | 1,566022258 |  |  |
|  | 3,815867332 | 1,750542855 |  |  |
|  | 2,369582728 | 1,871586044 |  |  |
|  | 2,730189014 | 2,159597945 |  |  |
|  | 2,79185678 | 2,336131278 |  |  |
|  | 2,953306732 | 2,395961113 |  |  |
|  | 2,630182946 | 2,40267477 |  |  |
|  | 2,831802933 | 2,31824392 |  |  |
| Malvasia C. a. |  |  |  |  |
|  | 2,562743547 | 1,043850896 |  |  |
|  | 1,188586811 | 1,127509233 |  |  |
|  | 1,157552854 | 1,02009797 |  |  |
|  | 1,235835465 | 1,322768618 |  |  |
|  | 0,956502919 | 1,397288532 |  |  |
|  | 0,707804924 | 1,445789985 |  |  |
|  | 0,890281513 | 1,616678145 |  |  |
|  |  |  |  |  |
| Fig.7A -Anth_RG x Total berry anthocyanins | X axis | Y axis | Statistical Elaboration | # samples |
| CULTIVAR | Total berry Anthocyanins | ANTH_RG | Linear regression / Polynomial model | 27 |
| Barbera | 0,003041379 | -0,074 |  |  |
|  | 0,007315862 | -0,106 |  |  |
|  | 0,0034944 | -0,1011 |  |  |
|  | 0,007154627 | -0,1076 |  |  |
|  | 0,013282759 | -0,1017 |  |  |
|  | 0,01323 | -0,1031 |  |  |
|  | 0,128184 | 0,0554 |  |  |
|  | 0,161238 | 0,0083 |  |  |
|  | 0,060735743 | 0,0438 |  |  |
|  | 0,6434379 | 0,2334 |  |  |
|  | 0,684265672 | 0,2395 |  |  |
|  | 0,4291875 | 0,1023 |  |  |
|  | 1,027404 | 0,0624 |  |  |
|  | 1,0548468 | 0,1409 |  |  |
|  | 0,94549901 | 0,1851 |  |  |
|  | 1,359317647 | 0,0389 |  |  |
|  | 1,47522297 | 0,0499 |  |  |
|  | 1,539977822 | 0,0466 |  |  |
|  | 1,363014925 | -0,0012 |  |  |
|  | 1,548361188 | 0,0239 |  |  |
|  | 1,380232836 | 0,0123 |  |  |
|  | 1,415948744 | 0,0232 |  |  |
|  | 1,757530746 | -0,0027 |  |  |
|  | 1,506491457 | 0,0148 |  |  |
|  | 1,727977723 | 0,01 |  |  |
|  | 1,803918806 | 0,0727 |  |  |
|  | 2,089108966 | 0,0522 |  |  |
|  |  |  |  |  |
| Ervi | 0,004432161 | -0,1141 |  |  |
|  | 0,009298218 | -0,0946 |  |  |
|  | 0,006741 | -0,1128 |  |  |
|  | 0,025106436 | -0,1115 |  |  |
|  | 0,013164179 | -0,1108 |  |  |
|  | 0,01323 | -0,1067 |  |  |
|  | 0,3515904 | 0,1413 |  |  |
|  | 0,361300299 | 0,0931 |  |  |
|  | 0,095404455 | 0,01 |  |  |
|  | 0,799897313 | 0,2252 |  |  |
|  | 0,6184836 | 0,1687 |  |  |
|  | 0,399948657 | 0,1423 |  |  |
|  | 1,398641791 | 0,1297 |  |  |
|  | 0,814294752 | 0,1709 |  |  |
|  | 0,797072239 | 0,171 |  |  |
|  | 1,78542 | 0,0802 |  |  |
|  | 1,539153 | 0,0409 |  |  |
|  | 1,668486567 | 0,0329 |  |  |
|  | 1,929566897 | 0,0471 |  |  |
|  | 1,670255172 | 0,0043 |  |  |
|  | 1,706748878 | 0,058 |  |  |
|  | 2,175516832 | 0,0693 |  |  |
|  | 1,799751724 | -0,026 |  |  |
|  | 2,040576238 | 0,0244 |  |  |
|  | 2,1027951 | -0,0555 |  |  |
|  | 2,005032414 | 0,0209 |  |  |
|  | 1,863885882 | -0,0048 |  |  |
|  |  |  |  |  |
| Malvasia R. | 0,053802 | -0,1162 |  |  |
|  | 0,036458911 | -0,1161 |  |  |
|  | 0,052355172 | -0,116 |  |  |
|  | 0,02260402 | -0,1163 |  |  |
|  | 0,0297675 | -0,1161 |  |  |
|  | 0,036794483 | -0,0913 |  |  |
|  | 0,038367 | 0,2156 |  |  |
|  | 0,035154 | 0,1171 |  |  |
|  | 0,025889552 | -0,0084 |  |  |
|  | 0,178364179 | 0,5068 |  |  |
|  | 0,133724623 | 0,3214 |  |  |
|  | 0,099261386 | 0,188 |  |  |
|  | 0,2682918 | 0,5557 |  |  |
|  | 0,173712 | 0,356 |  |  |
|  | 0,178794 | 0,4334 |  |  |
|  | 0,274659 | 0,5545 |  |  |
|  | 0,212499104 | 0,5297 |  |  |
|  | 0,188496 | 0,4556 |  |  |
|  | 0,237917647 | 0,6047 |  |  |
|  | 0,306847059 | 0,5088 |  |  |
|  | 0,270732414 | 0,5334 |  |  |
|  | 0,182574 | 0,5056 |  |  |
|  | 0,162823448 | 0,4712 |  |  |
|  | 0,143355882 | 0,4713 |  |  |
|  | 0,197004706 | 0,5047 |  |  |
|  | 0,184678806 | 0,5348 |  |  |
|  | 0,207262687 | 0,4353 |  |  |
|  |  |  |  |  |
| Fig.7b -Anth_RG x Total skin anthocyanins | X axis | Y axis | Statistical Elaboration | # samples |
| CULTIVAR | Total skin Anthocyanins | ANTH_RG | Linear regression / Polynomial model | 27 |
| Barbera | 0,040316212 | -0,074 |  |  |
|  | 0,084165226 | -0,106 |  |  |
|  | 0,050789748 | -0,1011 |  |  |
|  | 0,118134919 | -0,1076 |  |  |
|  | 0,172541064 | -0,1017 |  |  |
|  | 0,187874001 | -0,1031 |  |  |
|  | 1,185574769 | 0,0554 |  |  |
|  | 0,88424026 | 0,0438 |  |  |
|  | 7,11851202 | 0,2334 |  |  |
|  | 6,07757082 | 0,2395 |  |  |
|  | 9,77681015 | 0,0624 |  |  |
|  | 9,338958929 | 0,1409 |  |  |
|  | 9,179219554 | 0,1851 |  |  |
|  | 19,88199719 | 0,0389 |  |  |
|  | 15,46136921 | 0,0499 |  |  |
|  | 6,07757082 | 0,2395 |  |  |
|  | 14,14319305 | 0,0123 |  |  |
|  | 15,35747713 | 0,0466 |  |  |
|  | 13,39967803 | -0,0012 |  |  |
|  | 14,22815864 | 0,0239 |  |  |
|  | 14,14319305 | 0,0123 |  |  |
|  | 13,55063757 | 0,0232 |  |  |
|  | 15,88766699 | -0,0027 |  |  |
|  | 15,3467628 | 0,0148 |  |  |
|  | 18,25210402 | 0,01 |  |  |
|  | 20,0338697 | 0,0727 |  |  |
|  | 18,10576565 | 0,0522 |  |  |
|  |  |  |  |  |
| Ervi | 0,057760466 | -0,1141 |  |  |
|  | 0,110612924 | -0,0946 |  |  |
|  | 0,092963885 | -0,1128 |  |  |
|  | 0,291186416 | -0,1115 |  |  |
|  | 0,175437898 | -0,1108 |  |  |
|  | 0,198372176 | -0,1067 |  |  |
|  | 1,417953616 | 0,01 |  |  |
|  | 9,592475098 | 0,2252 |  |  |
|  | 10,33510285 | 0,1687 |  |  |
|  | 17,11731594 | 0,1297 |  |  |
|  | 0,175437898 | -0,1108 |  |  |
|  | 26,5762087 | 0,0471 |  |  |
|  | 22,49062615 | 0,0244 |  |  |
|  | 10,89318871 | 0,1709 |  |  |
|  | 10,96246872 | 0,171 |  |  |
|  | 23,40409097 | 0,0802 |  |  |
|  | 20,67281857 | 0,0409 |  |  |
|  | 18,06144031 | 0,0329 |  |  |
|  | 26,5762087 | 0,0471 |  |  |
|  | 21,52761576 | 0,0043 |  |  |
|  | 25,33890554 | 0,058 |  |  |
|  | 22,80053185 | 0,0693 |  |  |
|  | 21,60011571 | -0,026 |  |  |
|  | 22,49062615 | 0,0244 |  |  |
|  | 20,81615363 | -0,0555 |  |  |
|  | 24,13274576 | 0,0209 |  |  |
|  | 21,40396015 | -0,0048 |  |  |
|  |  |  |  |  |
| Malvasia R. | 0,48588979 | -0,1162 |  |  |
|  | 0,424493143 | -0,1161 |  |  |
|  | 0,445529919 | -0,116 |  |  |
|  | 0,192830077 | -0,1163 |  |  |
|  | 0,318547936 | -0,1161 |  |  |
|  | 0,471570506 | -0,0913 |  |  |
|  | 0,338327952 | 0,2156 |  |  |
|  | 0,373726785 | 0,1171 |  |  |
|  | 0,220359411 | -0,0084 |  |  |
|  | 2,884561507 | 0,5068 |  |  |
|  | 1,791638932 | 0,3214 |  |  |
|  | 1,282076658 | 0,188 |  |  |
|  | 2,414349007 | 0,5557 |  |  |
|  | 2,468722349 | 0,356 |  |  |
|  | 2,458597012 | 0,4334 |  |  |
|  | 2,349100411 | 0,5545 |  |  |
|  | 1,828887745 | 0,5297 |  |  |
|  | 2,032948264 | 0,4556 |  |  |
|  | 2,197095449 | 0,6047 |  |  |
|  | 1,690196286 | 0,5088 |  |  |
|  | 1,364136736 | 0,5334 |  |  |
|  | 2,657189325 | 0,5056 |  |  |
|  | 1,77343169 | 0,4712 |  |  |
|  | 1,90685965 | 0,4713 |  |  |
|  | 2,979772265 | 0,5047 |  |  |
|  | 2,387767577 | 0,5348 |  |  |
|  | 1,955348154 | 0,4353 |  |  |
|  |  |  |  |  |
| Fig.8A -FERARI x Total berry anthocyanins | X axis | Y axis | Statistical Elaboration | # samples |
| CULTIVAR | Total berry Anthocyanins | FERARI | Linear regression / Polynomial model | 27 |
| Barbera | 0,003041379 | -0,0704 |  |  |
|  | 0,007315862 | -0,077 |  |  |
|  | 0,0034944 | -0,0767 |  |  |
|  | 0,007154627 | -0,0771 |  |  |
|  | 0,013282759 | -0,0769 |  |  |
|  | 0,01323 | -0,0766 |  |  |
|  | 0,128184 | -0,0258 |  |  |
|  | 0,161238 | -0,0417 |  |  |
|  | 0,060735743 | -0,0158 |  |  |
|  | 0,6434379 | 0,6955 |  |  |
|  | 0,684265672 | 0,5761 |  |  |
|  | 0,4291875 | 0,3582 |  |  |
|  | 1,027404 | 1,354 |  |  |
|  | 1,0548468 | 1,166 |  |  |
|  | 0,94549901 | 1,024 |  |  |
|  | 1,359317647 | 1,341 |  |  |
|  | 1,47522297 | 1,539 |  |  |
|  | 1,539977822 | 1,579 |  |  |
|  | 1,363014925 | 1,642 |  |  |
|  | 1,548361188 | 1,477 |  |  |
|  | 1,380232836 | 1,502 |  |  |
|  | 1,415948744 | 1,395 |  |  |
|  | 1,757530746 | 1,54 |  |  |
|  | 1,506491457 | 1,477 |  |  |
|  | 1,727977723 | 1,644 |  |  |
|  | 1,803918806 | 1,653 |  |  |
|  | 2,089108966 | 1,496 |  |  |
|  |  |  |  |  |
| Ervi | 0,004432161 | -0,0771 |  |  |
|  | 0,009298218 | -0,0762 |  |  |
|  | 0,006741 | -0,0771 |  |  |
|  | 0,025106436 | -0,0771 |  |  |
|  | 0,013164179 | -0,0771 |  |  |
|  | 0,01323 | -0,077 |  |  |
|  | 0,3515904 | 0,3544 |  |  |
|  | 0,361300299 | 0,1206 |  |  |
|  | 0,095404455 | -0,0436 |  |  |
|  | 0,799897313 | 0,6356 |  |  |
|  | 0,6184836 | 0,5533 |  |  |
|  | 0,399948657 | 0,2846 |  |  |
|  | 1,398641791 | 1,339 |  |  |
|  | 0,814294752 | 1,05 |  |  |
|  | 0,797072239 | 1,162 |  |  |
|  | 1,78542 | 1,414 |  |  |
|  | 1,539153 | 1,49 |  |  |
|  | 1,668486567 | 1,507 |  |  |
|  | 1,929566897 | 1,449 |  |  |
|  | 1,670255172 | 1,514 |  |  |
|  | 1,706748878 | 1,354 |  |  |
|  | 2,175516832 | 1,504 |  |  |
|  | 1,799751724 | 1,633 |  |  |
|  | 2,040576238 | 1,318 |  |  |
|  | 2,1027951 | 1,529 |  |  |
|  | 2,005032414 | 1,47 |  |  |
|  | 1,863885882 | 1,592 |  |  |
|  |  |  |  |  |
| Malvasia R. | 0,053802 | -0,0771 |  |  |
|  | 0,036458911 | -0,0771 |  |  |
|  | 0,052355172 | -0,0771 |  |  |
|  | 0,02260402 | -0,0771 |  |  |
|  | 0,0297675 | -0,0771 |  |  |
|  | 0,036794483 | -0,0759 |  |  |
|  | 0,038367 | 0,0062 |  |  |
|  | 0,035154 | -0,043 |  |  |
|  | 0,025889552 | -0,0702 |  |  |
|  | 0,178364179 | 0,2965 |  |  |
|  | 0,133724623 | 0,0149 |  |  |
|  | 0,099261386 | 0,0052 |  |  |
|  | 0,2682918 | 0,678 |  |  |
|  | 0,173712 | 0,298 |  |  |
|  | 0,178794 | 0,2824 |  |  |
|  | 0,274659 | 0,747 |  |  |
|  | 0,212499104 | 0,5739 |  |  |
|  | 0,188496 | 0,402 |  |  |
|  | 0,237917647 | 0,836 |  |  |
|  | 0,306847059 | 0,7777 |  |  |
|  | 0,270732414 | 0,9263 |  |  |
|  | 0,182574 | 0,6641 |  |  |
|  | 0,162823448 | 0,6865 |  |  |
|  | 0,143355882 | 0,5673 |  |  |
|  | 0,197004706 | 0,5859 |  |  |
|  | 0,184678806 | 0,7435 |  |  |
|  | 0,207262687 | 0,7458 |  |  |
|  |  |  |  |  |
| Fig.8b -FERARI x Total skin anthocyanins | X axis | Y axis | Statistical Elaboration | # samples |
| CULTIVAR | Total skin Anthocyanins | FERARI | Linear regression / Polynomial model | 27 |
| Barbera | 0,003041379 | 0,040316212 |  |  |
|  | 0,007315862 | 0,084165226 |  |  |
|  | 0,0034944 | 0,050789748 |  |  |
|  | 0,007154627 | 0,118134919 |  |  |
|  | 0,013282759 | 0,172541064 |  |  |
|  | 0,01323 | 0,187874001 |  |  |
|  | 0,128184 | 1,185574769 |  |  |
|  | 1,506491457 | 15,3467628 |  |  |
|  | 0,060735743 | 0,88424026 |  |  |
|  | 0,6434379 | 7,11851202 |  |  |
|  | 0,684265672 | 6,07757082 |  |  |
|  | 1,363014925 | 13,39967803 |  |  |
|  | 1,027404 | 9,77681015 |  |  |
|  | 1,0548468 | 9,338958929 |  |  |
|  | 0,94549901 | 9,179219554 |  |  |
|  | 1,359317647 | 19,88199719 |  |  |
|  | 1,47522297 | 15,46136921 |  |  |
|  | 1,539977822 | 15,35747713 |  |  |
|  | 1,363014925 | 13,39967803 |  |  |
|  | 1,548361188 | 14,22815864 |  |  |
|  | 1,380232836 | 14,14319305 |  |  |
|  | 1,415948744 | 13,55063757 |  |  |
|  | 1,757530746 | 15,88766699 |  |  |
|  | 1,506491457 | 15,3467628 |  |  |
|  | 1,727977723 | 18,25210402 |  |  |
|  | 1,803918806 | 20,0338697 |  |  |
|  | 2,089108966 | 18,10576565 |  |  |
|  |  |  |  |  |
| Ervi | 0,004432161 | 0,057760466 |  |  |
|  | 0,009298218 | 0,110612924 |  |  |
|  | 0,006741 | 0,092963885 |  |  |
|  | 0,025106436 | 0,291186416 |  |  |
|  | 0,013164179 | 0,175437898 |  |  |
|  | 0,01323 | 0,198372176 |  |  |
|  | 0,009298218 | 0,110612924 |  |  |
|  | 0,797072239 | 10,96246872 |  |  |
|  | 0,095404455 | 1,417953616 |  |  |
|  | 0,799897313 | 9,592475098 |  |  |
|  | 0,6184836 | 10,33510285 |  |  |
|  | 1,670255172 | 21,52761576 |  |  |
|  | 1,398641791 | 17,11731594 |  |  |
|  | 0,814294752 | 10,89318871 |  |  |
|  | 0,797072239 | 10,96246872 |  |  |
|  | 1,78542 | 23,40409097 |  |  |
|  | 1,539153 | 20,67281857 |  |  |
|  | 1,668486567 | 18,06144031 |  |  |
|  | 1,929566897 | 26,5762087 |  |  |
|  | 1,670255172 | 21,52761576 |  |  |
|  | 1,706748878 | 25,33890554 |  |  |
|  | 2,175516832 | 22,80053185 |  |  |
|  | 1,799751724 | 21,60011571 |  |  |
|  | 2,040576238 | 22,49062615 |  |  |
|  | 2,1027951 | 20,81615363 |  |  |
|  | 2,005032414 | 24,13274576 |  |  |
|  | 1,863885882 | 21,40396015 |  |  |
|  |  |  |  |  |
| Malvasia R. | 0,053802 | 0,48588979 |  |  |
|  | 0,036458911 | 0,424493143 |  |  |
|  | 0,052355172 | 0,445529919 |  |  |
|  | 0,02260402 | 0,192830077 |  |  |
|  | 0,0297675 | 0,318547936 |  |  |
|  | 0,036794483 | 0,471570506 |  |  |
|  | 0,038367 | 0,338327952 |  |  |
|  | 0,035154 | 0,373726785 |  |  |
|  | 0,025889552 | 0,220359411 |  |  |
|  | 0,178364179 | 2,884561507 |  |  |
|  | 0,133724623 | 1,791638932 |  |  |
|  | 0,099261386 | 1,282076658 |  |  |
|  | 0,2682918 | 2,414349007 |  |  |
|  | 0,173712 | 2,468722349 |  |  |
|  | 0,178794 | 2,458597012 |  |  |
|  | 0,274659 | 2,349100411 |  |  |
|  | 0,212499104 | 1,828887745 |  |  |
|  | 0,188496 | 2,032948264 |  |  |
|  | 0,237917647 | 2,197095449 |  |  |
|  | 0,306847059 | 1,690196286 |  |  |
|  | 0,270732414 | 1,364136736 |  |  |
|  | 0,182574 | 2,657189325 |  |  |
|  | 0,162823448 | 1,77343169 |  |  |
|  | 0,143355882 | 1,90685965 |  |  |
|  | 0,197004706 | 2,979772265 |  |  |
|  | 0,184678806 | 2,387767577 |  |  |
|  | 0,207262687 | 1,955348154 |  |  |
